# Setdb1 safeguards proper differentiation of adult intestinal stem cells by controlling chromatin accessibility and transcriptome variability

**DOI:** 10.1016/j.isci.2026.116731

**Published:** 2026-07-09

**Authors:** Ioanna Peraki, Liwei Zhang, Dimitris Botskaris, Marianna Stagaki, Ioannis K. Deligiannis, Haroula Kontaki, Elena Deligianni, Ioannis Giannoulakis, Orsalia Hazapis, Matthieu D. Lavigne, Celia P. Martinez-Jimenez, Iannis Talianidis

**Affiliations:** 1 Institute of Molecular Biology & Biotechnology, Foundation for Research and Technology, Hellas (IMBB-FORTH), Heraklion, Crete, Greece; 2 Department of Biology University of Crete, Heraklion, Crete, Greece; 3 Helmholtz Pioneer Campus (HPC), Helmholtz Zentrum Munchen, Neuherberg, Germany; 4 TUM School of Medicine, Technical University of Munich, Munich, Germany; 5 Institute of Biotechnology and Biomedicine (BIOTECMED), Department of Biochemistry and Molecular Biology, University of Valencia, Burjassot, Spain

**Keywords:** intestinal stem cells, Setdb1, heterochromatin, differentiation, transcriptome variability, gene regulatory networks, virtual knock-out

## Abstract

The histone methylase Setdb1 plays a pivotal role in embryonic stem cell maintenance and developmental lineage specification. However, its function in adult stem cells remains elusive. Here we show that the conditional inactivation of *Setdb1* in Lgr5^+^ intestinal stem cells alters the transcriptional programs of the progeny cell types and results in increased cell-to-cell transcriptional variability. Loss of Setdb1 blocked differentiation toward the absorptive enterocyte lineage, while the secretory cell types were only marginally affected due to the activation of alternative developmental trajectories. *Setdb1* inactivation did not affect global H3K9 methylation at large heterochromatin domains but led to altered distribution of transposase-accessible chromatin regions, aberrant exposure of transcription factor binding sites, and premature activation of differentiation-specific genes. The results demonstrate that Setdb1 regulates intestinal stem cell differentiation by fine-tuning chromatin accessibility in open euchromatin regions, thereby controlling transcriptional variability between cells.

## Highlights

• Setdb1 prevents premature gene activation during intestinal stem cell differentiation
• Setdb1 controls chromatin accessibility in gene-rich regions
• Setdb1 is a global epigenetic gatekeeper that stabilizes developmental transitions
• Cell-to-cell transcriptome variability is a hallmark of differentiation transitions

## Graphical abstract

**Figure:**
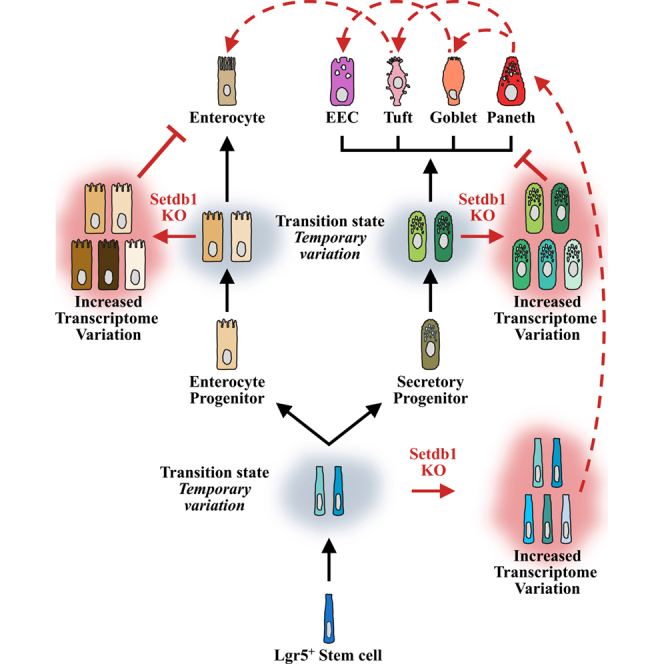

## Teaser

Molecular biology; Epigenetics; Stem cells research; Developmental biology

## Introduction

A significant proportion of the eukaryotic genome is organized in highly compacted structures, called “constitutive” heterochromatin, which plays a fundamental role in maintaining genome stability via inhibiting the activity of mobile transposable elements, silencing repetitive and retroviral sequences, and ensuring proper chromosome segregation.^1^^,^^2^^,^^3^ Heterochromatin organization is also detectable in specific gene areas, forming locally closed but reversible structures, called “facultative” heterochromatin, which contributes to condition-specific transcriptional repression.^4^ Nucleosomes at heterochromatin are densely modified by methylations of histone 3 (H3K9Me_2/3_ and H3K27Me_3_) or histone 4 (H4K20Me_3_).^2^ H3K9 di- and trimethylation at major blocks of repeat-rich heterochromatin, such as centromeric and telomeric regions, is catalyzed by three major methyltransferases, Suv39h1, Suv39h2, and Setdb1.^5^^,^^6^^,^^7^^,^^8^

Dynamic changes in heterochromatin play crucial roles in cell lineage specification during early embryonic development, stem cell maintenance, and differentiation.^9^^,^^10^^,^^11^^,^^12^^,^^13^ The function of the three main H3K9 methylating enzymes in heterochromatin formation displays considerable redundancy, as the full eradication of H3K9Me_2/3_ marks can only be achieved in double and triple knockout cells.^5^^,^^7^^,^^9^^,^^14^ Despite this redundancy, genetic inactivation of the individual enzymes often leads to aberrant phenotypes, such as impaired differentiation, genome instability, viral mimicry, necroptosis, or cancer.^3^^,^^10^^,^^15^

Setdb1 has been postulated to influence gene expression via multiple mechanisms. These include control of constitutive heterochromatin spreading, formation of facultative heterochromatin,^6^ silencing of LTR retrotransposon insertions^5^ and methylation of transcription factors (TFs) and signaling regulators.^16^^,^^17^^,^^18^ Setdb1 is also important for the formation of poised enhancers, where it cooperates with NSD1 methylase to create nucleosomes with dual histone modification marks, H3K9_Me_/H3K36_Me_.^19^

The role of Setdb1 in embryonic stem cells (ESCs) has been extensively studied.^6^^,^^12^^,^^13^^,^^20^ Apart from its pivotal role in maintaining genome stability via silencing endogenous retroviral elements,^3^ Setdb1 has been shown to cooperate with PRC2 to suppress developmental regulators^20^ and contribute to the transition of facultative heterochromatin to constitutive heterochromatin during differentiation. Consistent with this, loss of Setdb1 in ESCs and in more differentiated cells often leads to altered transcription programs, suggesting that Setdb1-mediated silencing is important for the regulation of tissue-specific gene expression patterns.^10^^,^^12^ Furthermore, H3K9 methylation has been shown to play a role in maintaining high levels of DNA methylation in specific retroviral elements, demonstrating cell type-specific effects of Setdb1-mediated H3K9 methylation in silencing retroviral transcripts.^21^

Multipotency of adult stem cells, similar to the maintenance of pluripotency in ES cells, is governed by the function of transcriptional regulators, which activate an array of genes characteristic of the stem cell phenotype and, in parallel, repress genes conferring the differentiated cell phenotype.^22^ Recent studies have indicated that Setdb1-mediated H3K9 methylation may be involved in the specification of multipotent phenotypes in different adult tissues, via regulating the binding of the architectural factor CTCF and consequently CTCF-dependent formation of genomic loops^23^ or by preventing the binding of developmental TFs to non-canonical sites, which ensures proper differentiation of hematopoietic stem cells.^24^

To further explore the developmental role of Setdb1 in adult stem cell differentiation, we investigated its function in Lgr5^+^ intestinal stem cells. Lgr5^+^ stem cells are found at the base of the intestinal crypts, embedded into a microenvironment of specialized cells, which provide the necessary repertoire of signaling molecules, such as Wnt, Notch, BMP, to regulate self-renewal and differentiation.^25^ Lgr5^+^ stem cells give rise to transit amplifying (TA) cells and proliferating progenitors of secretory or absorptive lineages that subsequently differentiate to each principal cell type of the intestinal epithelium, including Paneth cells, Goblet cells, enterocytes, enteroendocrine (EEC) cells, and Tuft cells.^25^^,^^26^

In this study, using lineage tracing and single-cell RNA-sequencing (scRNA-seq) combined with single-cell ATAC-sequencing (scATAC-seq) approaches, we demonstrate that Setdb1 is required for the proper differentiation of Lgr5^+^ stem cells into distinct intestinal epithelial cell types. The results reveal an unexpected function of Setdb1 in open chromatin domains, which contributes to the tight control of the expression of linked genes, and plays a fundamental role in shaping cell identity in the rapidly renewing intestinal epithelium.

## Results

### *Setdb1* inactivation results in defective differentiation of Lgr5^+^ cells in the intestinal epithelium

**Figure 1:**
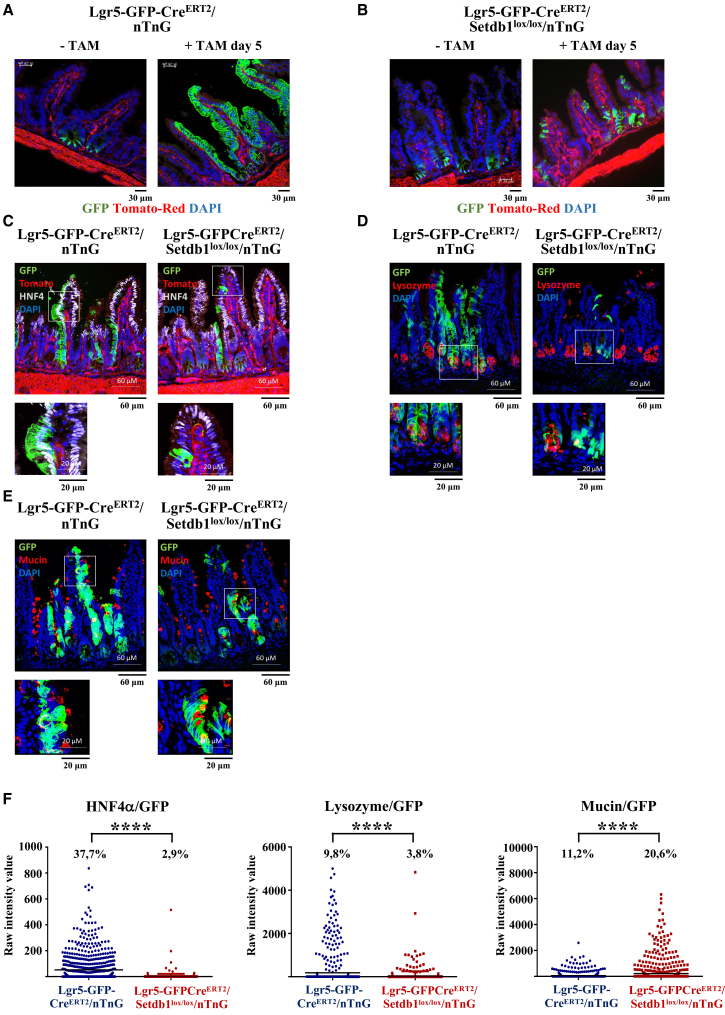
Setdb1 is required for the differentiation of Lgr5^+^ stem cells to intestinal epithelial cell types (A and B) Representative fluorescent images from the ileum of Lgr5-GFP-Cre^ERT2^/nTnG mice, expressing wild-type Setdb1 mRNA (A), and Lgr5-GFP-Cre^ERT2^/Setdb1^lox/lox^/nTnG mice, carrying floxed alleles of Setdb1 (B) that were treated with tamoxifen (+TAM) or with solvent (-TAM) for 5 days *n* = 4 mice. (C–E) Representative immunofluorescence staining of ileal epithelium with antibodies recognizing the mature Enterocyte marker HNF4α (C), the Paneth cell marker Lysozyme (D), and the Goblet cell marker Mucin (E). Bottom panels correspond to zoom-in images of the indicated areas. *n* = 4 mice. (F) Estimation of HNF4a^+^ enterocytes, Lysozyme^+^ Paneth cells, and Mucin^+^ Goblet cells in GFP^+^ cell populations from Tamoxifen-treated Lgr5-GFP-Cre^ERT2^/nTnG and Lgr5-GFP-Cre^ERT2^/Setdb1^lox/lox^/nTnG. Intestinal epithelial cells from the mice were isolated and plated onto glass coverslips. The coverslips were stained with antibodies against HNF4a, Lysozyme and Mucin2. The graphs show High content Microscopy (HCM) measurements of individual cells double stained with the above markers and with GFP antibody from 786 cells in HNF4a/GFP-stained samples, from 802 cells in Lysozyme/GFP samples and 819 cells in Mucin/GFP-stained samples. Only cells with sum-intensity above background and with proper intracellular localization were counted. Numbers indicate the percentage of cells with staining intensity above threshold. ∗∗∗∗*p* < 0.0001, determined by Student’s *t* test.

To investigate the function of Setdb1 in the self-renewal and differentiation properties of the intestinal Lgr5^+^ adult stem cells, we crossed mice carrying Lgr5-GFP-Cre^ERT2^ knock-in allele^25^ with mice carrying floxed exon-3 *Setdb1* alleles (Figures S1A and S1B). In these mice, Lgr5^+^ intestinal stem cells are labeled by GFP, and upon tamoxifen treatment, *Setdb1* is selectively inactivated in GFP-expressing Lgr5^+^ stem cells (Figures S1E and S1F). To follow the fate of Lgr5^+^ cells during differentiation, we crossed Lgr5-GFP-Cre^ERT2^ and Lgr5-GFP-Cre^ERT2^/Setdb1^lox/lox^ mice with ROSA (CAG-tdTomato∗, EGFP∗)Ees mice (abbreviated as nTnG), carrying the dual reporter of tomato-red and EGFP in the ROSA locus (Figures S1C and S1D). In these mice, after tamoxifen treatment, GFP is permanently expressed in both Lgr5^+^ stem cells and their progeny, due to the irreversible loss of the tdTomato gene. As expected, GFP-labeled cells were localized only at the bottom of the crypts in mock-treated cells (Figure 1A). In agreement with previous reports, which showed that Lgr5^+^ cells can give rise to all intestinal epithelial cell types,^25^ we observed GFP-labeled cells over the entire crypt and villi in a continuous fashion five days after tamoxifen treatment (Figure 1A). The labeled strip of epithelial cells originating from the bottom of the crypts was missing in some areas, which is explained by the known mosaic expression of the Lgr5 knock-in allele caused by random silencing.^25^^,^^26^

In contrast, *Setdb1* inactivation in Lgr5^+^ cells resulted in only a few GFP-labeled cells with scattered distribution along the crypt-villus axis and some accumulation of the labeled cells at the bottom of the crypts (Figure 1B). Double-immunostaining assays with antibodies against the enterocyte marker HNF4α, the Paneth cell marker Lyzozyme (Lyz), and the Goblet cell marker Mucin (Muc), revealed a strong reduction of the HNF4α/GFP double-positive cells in *Setdb1*-KO mice (Figures 1C and 1F). Lyz/GFP double-positive cells were reduced to a lesser degree (Figures 1D and 1F), while Muc/GFP double-positive cells have almost doubled (Figures 1E and 1F). The above staining patterns suggest that Setdb1-deficiency leads to a major defect of Lgr5^+^ stem cell differentiation toward epithelial lineages, especially to enterocytes.

Independent evidence for the requirement of Setdb1 in Lgr5^+^ stem cell differentiation was provided by *in vitro* organoid formation assays. Previous studies have established that single Lgr5^+^ stem cells can differentiate *in vitro* and give rise to self-organizing organoid structures, which contain all the intestinal epithelial cell types.^27^ We isolated GFP-expressing cells from the ileum of Lgr5-GFP-Cre^ERT2^-nTnG and Lgr5-GFP-Cre^ERT2^/Setdb1^lox/lox^/nTnG mice by FACS-sorting and cultured them in organoid culture conditions. From the day of seeding, the cells were treated with tamoxifen, and the formation of GFP-labeled 3D organoids was monitored. As shown in Figure S1G, 10-day after tamoxifen treatment, complex 3D organoid structures with several crypt-like domains were developed from Lgr5-GFP-Cre^ERT2^-nTnG cells, with the majority of the cells being GFP positive. In contrast, cells from the Lgr5-GFP-Cre^ERT2^/Setdb1^lox/lox^/nTnG mice developed simpler structures, and the cells displayed heterogeneous GFP-label intensity (Figure S1G). Importantly, when the cells from the GFP-labeled organoids were subjected to a second passage, only the cultures derived from normal *Setdb1* allele-containing stem cells gave rise to fully developed 3D GFP-labeled organoid structures. Organoids derived from *Setdb1*-KO cells generated small cellular assemblies with spherical morphology and near background-level GFP fluorescence (Figure S1H).

### Setdb1 requirement for proper intestinal epithelial cell specification reveals alternative unconventional trajectories toward secretory and endocrine-related cell lineages

**Figure 2:**
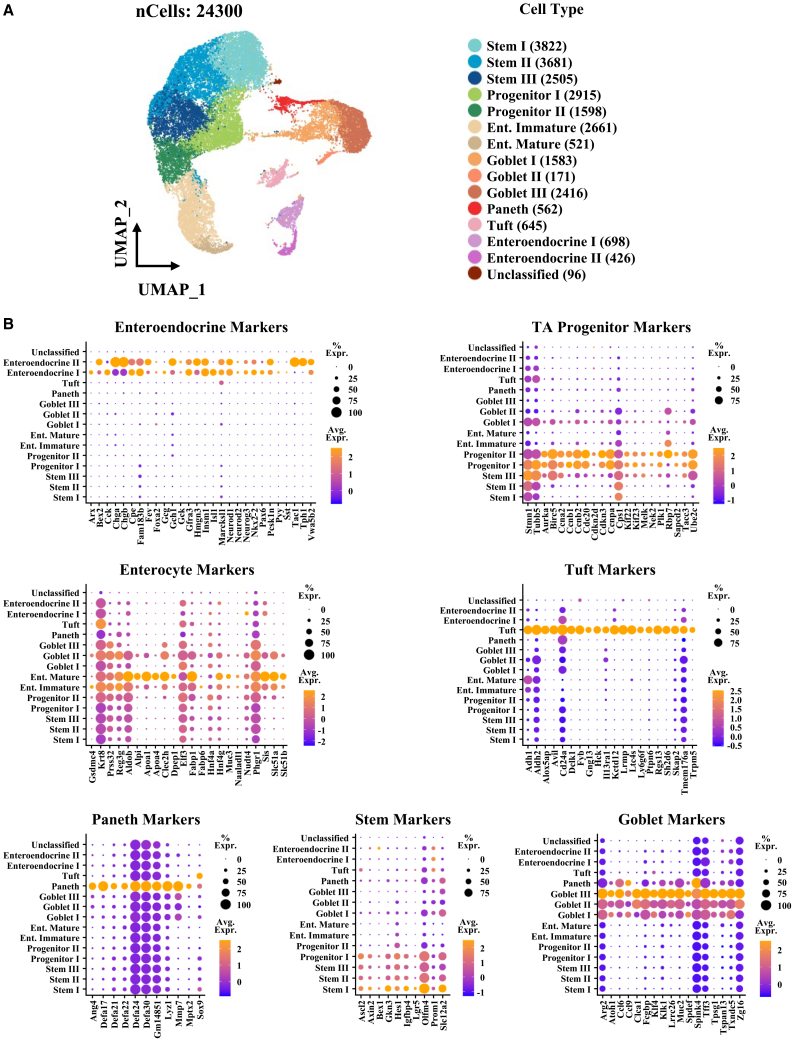
Identification of intestinal epithelial cell types (A) UMAP projection of 24.300 single cells (merged datasets from 2 biological replicates) identifies cell types based on the expression of known marker genes. The color codes of the annotated cell types are shown at right. (B) Dot blot analysis of marker gene expression in the different cell clusters.

To determine whether Setdb1 affects Lgr5^+^ stem cell differentiation at early or late stages, we performed single-cell RNA-seq experiments after FACS sorting of GFP^+^ intestinal epithelial cells from Lgr5-GFP-Cre^ERT2^ and Lgr5-GFP-Cre^ERT2^/Setdb1^lox/lox^ mice (hereby named Lgr5^+^WT and Lgr5^+^Setdb1KO, respectively), five days after tamoxifen treatment. From two independent biological replicates, a total of 10,598 Lgr5^+^WT cells and 13,702 Lgr5^+^Setdb1KO high-quality cells with an average of 2550 genes per cell were analyzed. After data merging and integration, unsupervised graph clustering was used to partition the cells into 15 clusters, and the cell types were annotated using previously described marker gene signatures.^28^^,^^29^ The results were visualized by Uniform Manifold Approximation and Projection (UMAP) (Figures 2A and 2B). About 8752 cells, corresponding to 82% of the total, expressed GFP mRNA. These cells were identified as Lgr5^+^ stem cells (Stem-I-II-III), early progenitors including TA cells (Progenitor-I-II-S), and potential lineage-specified progenitors (Enterocyte immature, Goblet-I and EEC-I). The remaining 18% of the cells corresponded to mature enterocytes, Goblet cells, Paneth cells, Tuft cells, and EEC cells, whose presence is explained by the gating strategy used and the incomplete enrichment of the Lgr5^+^GFP^+^ cell population by our FACS sorting.

We have included the latter “co-purifying” non-GFP^+^ cell clusters in our comparative transcriptome analyses, as they provided useful information for investigating Setdb1’s role in transcriptional transitions that define lineage trajectories during differentiation.

Regarding the stem cell compartment, each of the three distinct stem cell populations expressed pan-stem cell markers (Figures S2A–S2C) along with their corresponding cluster-specific gene signatures. The transcriptome of Stem-I cells is characteristic of the low cycling Lgr5^+^ stem cells, while the Stem-II cluster corresponds to the recently described MHCII-expressing antigen-presenting stem cell population.^30^^,^^31^ Stem-III cells represent a more proliferative cell population (Figures S2A–S2C).

The transcriptome profiles of the cells termed Progenitors revealed two main, distinct cell clusters. These cells correspond to the more proliferative downstream cell types, including +4 cells and TA cells,^29^^,^^32^ which correspond to precursors for secretory lineages (Progenitor-I) and for enterocytes (Progenitor-II) (Figures S2D and S2E). Furthermore, transcription profile analysis identified three Goblet cell populations (Figures S3A–S3C) and two EEC cell populations (Figures S3D and S3E). The transcriptome of Goblet-I and EEC-I cells suggests that they represent lineage-specified precursors of the more mature Goblet-II and III and EEC-II cells, respectively (Figure S3).^29^^,^^33^

**Figure 3:**
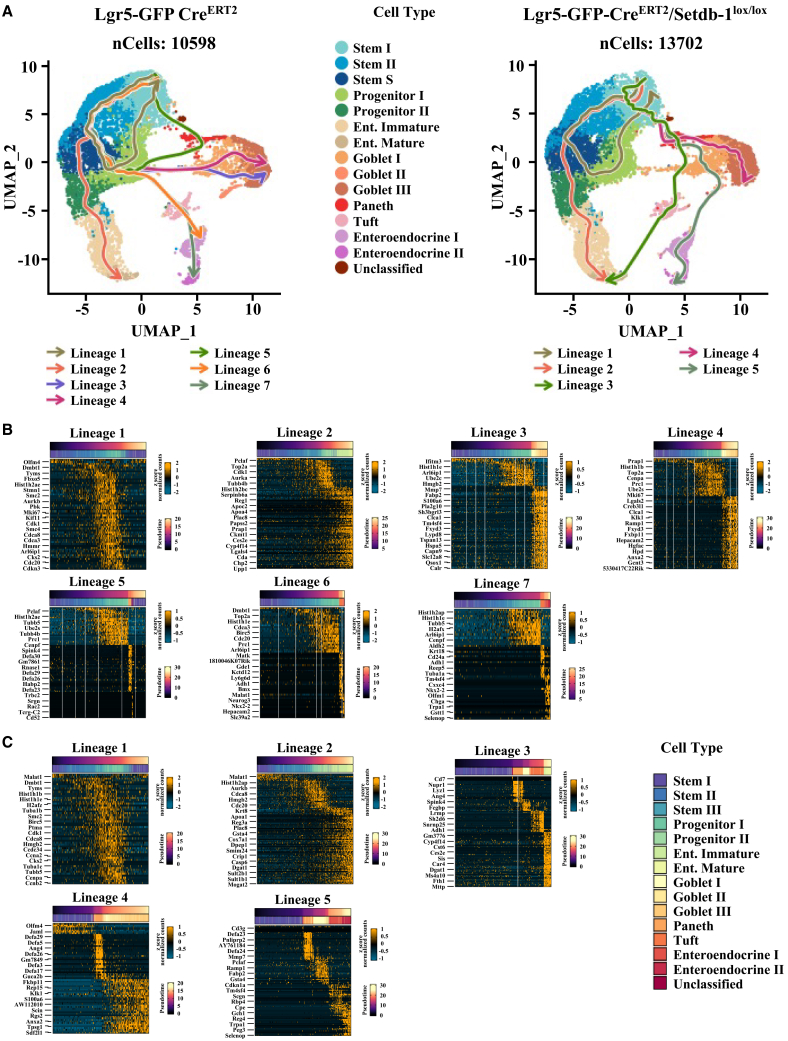
Dynamic transitions and alternative differentiation trajectories of epithelial cell types in Setdb1-deficient cells (A) UMAP projection of RNA velocities in single cells from Lgr5-GFP-Cre^ERT2^ (left panel) and Lgr5-GFP-Cre^ERT2^/Setdb1^lox/lox^ (right panel) mice after 5 days of tamoxifen treatment. Arrows indicate lineage trajectories from pseudotemporal inference, starting from the Stem-I cell cluster. The color codes corresponding to the different cell clusters are indicated in the middle. (B and C) Gene expression heatmap of differentially expressed genes of the displayed cell lineages plotted along velocity-based pseudotime starting from the Stem-I cell cluster. Panels in (B) show data from Lgr5-GFP-Cre^ERT2^ mice expressing the normal allele of Setdb1, while panels in (C) show data from tamoxifen-treated Lgr5-GFP-Cre^ERT2^/Setdb1^lox/lox^ mice, expressing the mutant allele of Setdb1. Top bars above the heatmaps indicate the pseudotemporal axis. The bars below indicate corresponding cell identity across the inferred trajectory, according to the color code. Color bars at the right show *Z* score normalized relative mRNA read counts (upper bar) and relative pseudotimes (lower bars).

To delineate the differentiation dynamics of the intestinal epithelial cell types, we performed pseudotemporal reconstruction of the transcriptional changes^34^^,^^35^ and mapped seven distinct continuous trajectories in cells from Lgr5-GFP-Cre^ERT2^ (control) mice (Figures 3A and 3B). As expected, each of the seven lineages originating from Stem-I cells gave rise to all known intestinal epithelial cell types. The intermediate cellular states in the different lineages revealed interesting cell fate dynamics. Lineage-1 represents a Stem-I cell self-renewal path through Stem-II, Stem-III, and Progenitor-I cellular states. Lineage-2 is the sole differentiation trajectory for the generation of mature enterocytes via Stem-II, Stem-III, Progenitor-II, and Immature Enterocyte cellular states. The hierarchical path from Stem-I to Stem-II, Stem-III, and Progenitor-I cells is common in Lineages 3, 4, 5, 6, and 7, which end up in Goblet cells (Lineages 3 and 4), in Paneth cells (Lineage-5), and EEC cells (Lineages 6–7). Interestingly, for Lineage-5 that gives rise to Paneth cells, the cell trajectory analysis reconstructs a differentiation path connecting Paneth cells back to the Stem-I cluster, supporting the possibility that de-differentiation of Paneth cells may also be involved in Stem-I self-renewal, as indicated in a previous report.^36^ Similarly, the trajectory topology for Lineages 6 and 7 shows EEC clusters as the final differentiation stage, placing the Tuft cell cluster in an intermediate stage within the differentiation process. These results indicate that Tuft cell clusters might represent an upstream and hierarchical connection between the processes regulating the differentiation of these cell types.

Analyses of *Setdb1-*KO cells from Lgr5-GFP-Cre^ERT2^/Setdb1^lox/lox^ mice revealed major alterations of the above trajectories (Figures 3A and 3C). While Lineage-1 and Lineage-2, specifying the Stem-I self-renewal and enterocyte differentiation paths, were not altered, the other trajectories specifying secretory cell types (Lineages 3, 4, 5, 6, 7) were lost (Figures 3A and 3C). In contrast, three new lineages with aberrantly ordered paths appeared, connecting Stem-I cells directly to Paneth cells and branching either toward Tuft cells and enterocytes through Goblet-I cells (KO-Lineage 3), or toward EEC-I and EEC-II cells through Goblet-II cell cluster (KO-Lineage-5), or toward Goblet-III cells through Goblet-II cell cluster (KO-Lineage-4).

**Figure 4:**
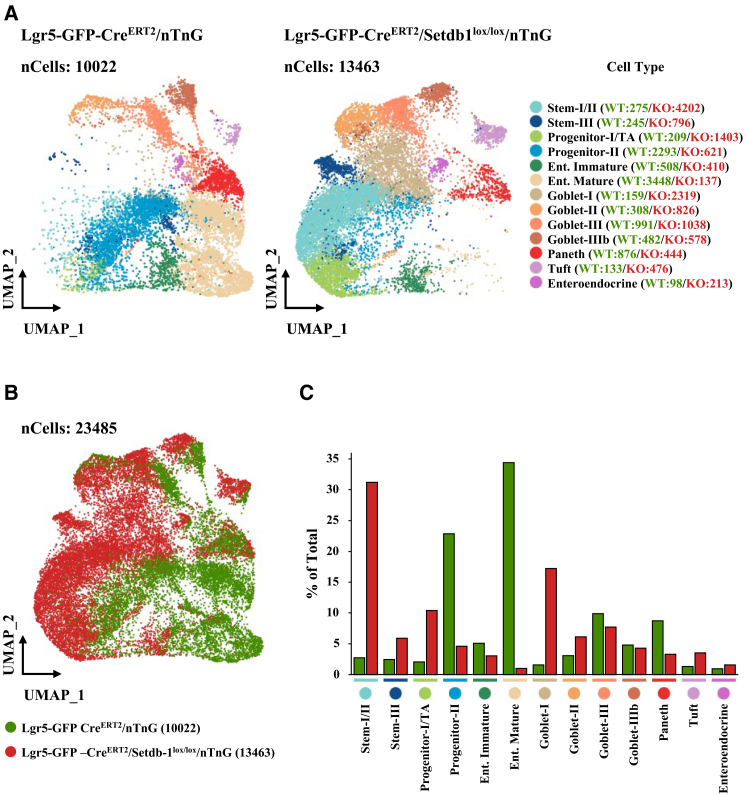
Lineage tracing of individual Lgr5^+^ progeny epithelial cells reveals defective differentiation of *Setdb1*-deficient Lgr5^+^ stem cells (A) UMAP projections of cell clusters obtained from FACS-sorted GFP^+^ cells isolated from tamoxifen-treated Lgr5-GFP-Cre^ERT2^/nTnG (left panel) and Lgr5-GFP-Cre^ERT2^/Setdb1^lox/lox^/nTnG mice (right panel). The color codes and the number of cells identified in the different cell clusters are indicated at the right. (B) Aligned UMAP plots of cells detected in samples from tamoxifen-treated Lgr5-GFP-Cre^ERT2^/nTnG (green dots) and Lgr5-GFP-Cre^ERT2^/Setdb1^lox/lox^/nTnG (red dots) mice. (C) Comparison of the percentages of cells corresponding to the different clusters in tamoxifen-treated Lgr5-GFP-Cre^ERT2^/nTnG (green columns) and Lgr5-GFP-Cre^ERT2^/Setdb1^lox/lox^/nTnG (red columns) mice.

While transcriptome-based trajectories accurately recapitulate the order of the consecutive differentiation stages,^34^ they do not provide a quantitative and comparative assessment of the actual extent of the operation of the stable or altered differentiation paths in specific conditions. Therefore, we further analyzed the functional importance of the above trajectories by performing single-cell transcriptome analyses in lineage-traced Lgr5^+^ cells, taking advantage of the fact that following tamoxifen treatment of Lgr5-GFP-Cre^ERT2^/nTnG and Lgr5-GFP-Cre^ERT2^-nTnG/Setdb1^lox/lox^/nTnG mice, only Lgr5^+^ cell-derived descendant cell populations acquire GFP expression, which can be isolated by FACS sorting. In agreement with the immunostaining results in Figure 1, five days after tamoxifen treatment, we could detect all the known intestinal epithelial cell types in the control Lgr5-GFP-Cre^ERT2^-nTnG mice (Figure 4A). In contrast, in Setdb1-KO cells from Lgr5-GFP-Cre^ERT2^-nTnG/Setdb1^lox/lox^ mice, a dramatic increase in the number and the proportion of Stem-I cells was observed along with a major decrease in the number and proportion of mature enterocytes (Figures 4A–4C). A moderate increase of Goblet, Tuft, and EEC cells was also observed, with the precursor Goblet-I population increasing the most. The number of Paneth cells in the *Setdb1-KO* Lgr5^+^ descendants was nearly halved.

The accumulation of Stem-I/II, Progenitor I/TA, and the early Goblet-I cells suggests that Setdb1-deficiency leads to differentiation inhibition rather than causing massive proliferation defects or cell death phenomena at the early time point of 5 day following Tamoxifen injection. Consistent with this, we observed only minor changes in the percentage of cells stained positively by the proliferation marker Ki67 or the apoptosis marker Caspase-3 antibodies, as well as in cell cycle distribution across different clusters or in crypt-villus ratios between wild type and *Setdb1*-KO cells (Figure S4).

Taken together, the above results demonstrate that Setdb1 deficiency results in a major block of Lgr5^+^ stem cell differentiation from the very early steps. The Setdb1 function is fully required for enterocyte differentiation and for the progression of the Progenitor-I cell population into other secretory and endocrine-related cell types. Nevertheless, these latter cell types can be generated directly from Lgr5^+^ Stem-I cells, via the activation of alternative differentiation trajectories.

### Setdb1 safeguards proper differentiation of Lgr5^+^ stem cells by preventing uncontrolled expansion of open accessible chromatin domains and premature aberrant gene activation

Previous studies using Villin-Cre^ERT2^ transgene, which inactivates *Setdb1* in all intestinal epithelial cell types, have demonstrated a noticeable induction of endogenous retrovirus expression, which triggered a viral mimicry and activated antiviral immune responses, culminating in inflammation and necroptosis.^37^^,^^38^ In our experimental setup, where *Setdb1* inactivation is restricted to Lgr5^+^ stem cell population and the analyses were performed as early as 5 days after Cre transgene activation, we failed to detect a massive increase of retroviral transcripts by bulk RNA-seq assay or the activation of innate immunity-related or necroptosis-related genes in a substantial number of cells by scRNA-seq (Figures S5A and S5B). Although retroviral silencing is likely to occur at later time points, the above result suggests that the observed early differentiation defect is rather the result of a direct function of Setdb1 in the regulation of differentiation-specific genes at early stem cell states.

**Figure 5:**
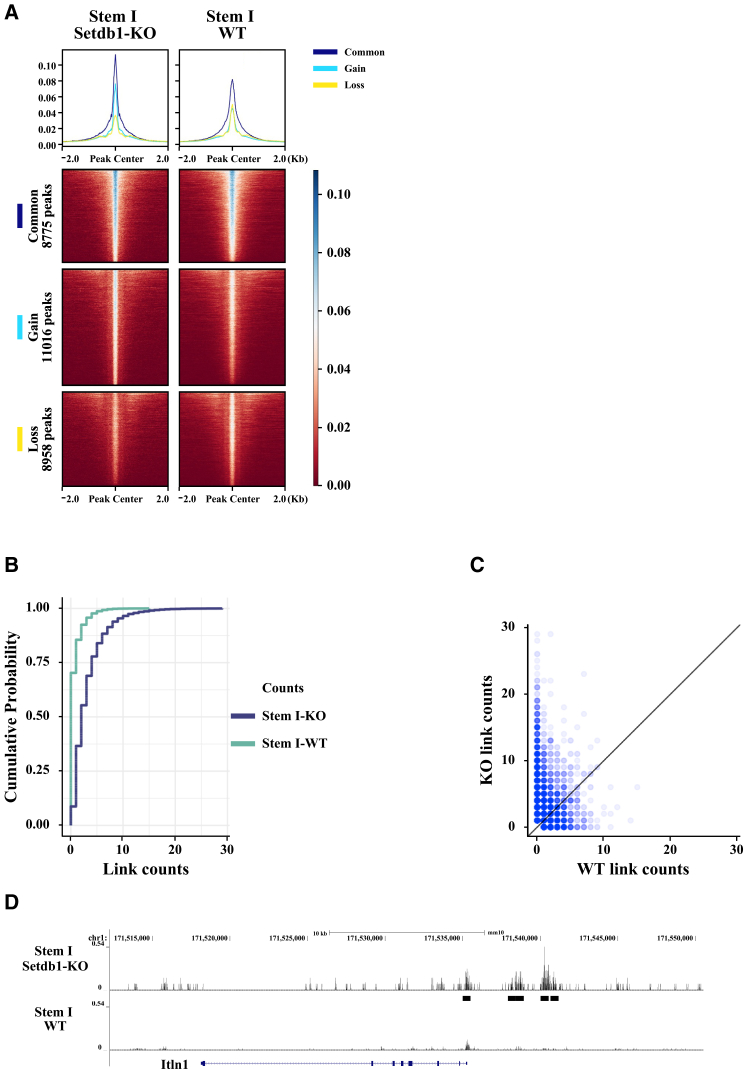
Reorganization of transposase accessible chromatin areas in *Setdb1*-deficient intestinal stem cells (A) Signal intensity heatmaps and average distribution profiles (top panels) of scATAC-seq peaks in the Stem-I cell cluster from tamoxifen-treated Lgr5-GFP-Cre^ERT2^ (WT) mice and Lgr5-GFP-Cre^ERT2^/Setdb1^lox/lox^ (*Setdb1*-KO) mice. Read distribution at the indicated regions around the center of ATAC-seq peaks is shown. ATAC-seq peaks were clustered according to their presence in both wild-type and *Setdb1*-KO cells (Common peaks), in *Setdb1*-KO cells only (Gained peaks), and in wild-type cells only (Lost peaks). (B) Quantitative comparisons of scATAC-seq peak variations in the Stem-1 cell cluster of tamoxifen-treated Lgr5-GFP-Cre^ERT2^ (WT) mice and Lgr5-GFP-Cre^ERT2^/Setdb1^lox/lox^ (*Setdb1*-KO) mice. The graph shows the number of ATAC-seq peaks linked to genes and the cumulative probability of their occurrence. (C) Scatterplot presentation of the peak-to-gene link counts in the Stem-1 cell cluster of tamoxifen-treated Lgr5-GFP-Cre^ERT2^ (WT) mice and Lgr5-GFP-Cre^ERT2^/Setdb1^lox/lox^ (*Setdb1*-KO) mice. Note the higher number of counts above the baseline in *Setdb1*-KO cells. (D) Genome Browser profile of normalized ATAC-seq reads and peaks-linked-to-genes (black bars) at the genomic region encompassing the aberrantly activated Itln-1 gene in tamoxifen-treated Lgr5-GFP-Cre^ERT2^ mice (WT) and in Lgr5-GFP-Cre^ERT2^/Setdb1^lox/lox^ mice (*Setdb1*-KO). Arrows indicate ATAC-seq peaks gained in *Setdb1*-deficient cells.

**Figure 6:**
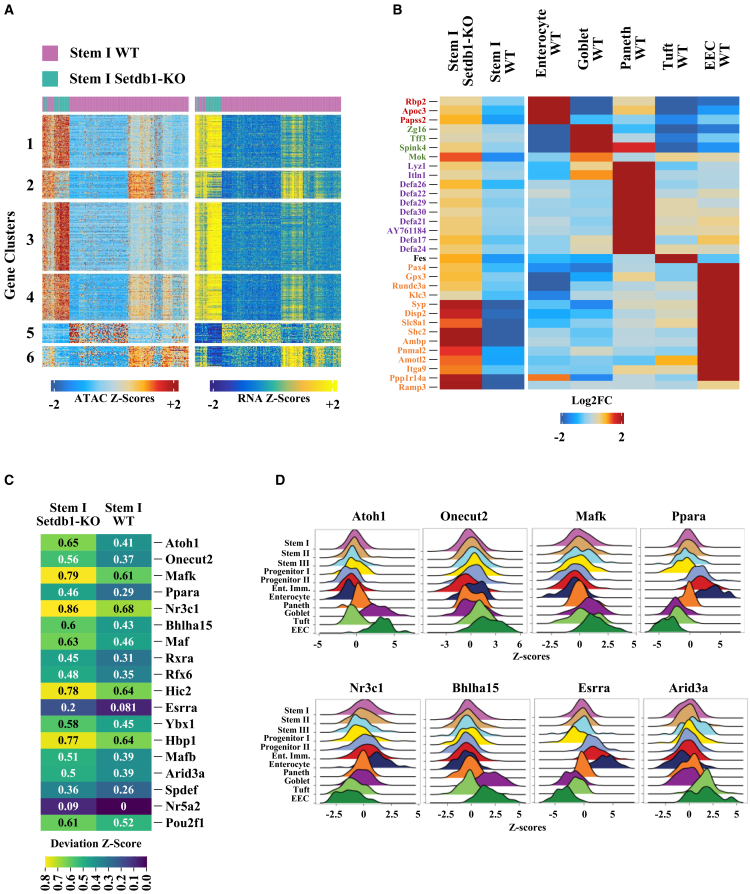
Aberrant activation of genes in *Setdb1*-deficient Lgr5^+^ stem cells (A) Heatmaps and corresponding clustering of integrated RNA signals (panel at right) and normalized scATAC-seq signals (left panel) of the 4890 ATAC-seq peaks linked to 3634 unique genes in Stem-I cell population from tamoxifen-treated Lgr5-GFP-Cre^ERT2^ (WT; purple) and Lgr5-GFP-Cre^ERT2^/Setdb1^lox/lox^ (*Setdb1*-KO; green) mice. The six gene clusters are separated by space. The distribution of the total ATAC-seq peaks and linked to unique genes between the clusters is as follows: Cluster 1: 1097 peaks/815 genes; Cluster 2: 578 peaks/460 genes; Cluster 3: 1415 peaks/1001 genes; Cluster 4: 963 peaks/748 genes; Cluster 5: 409 peaks/313 genes; Cluster 6: 428 peaks/297 genes. Note the strong correlation between ATAC-seq signal and RNA levels in all of the gene clusters and the *Setdb1*-KO-specific increased chromatin accessibility and RNA expression in gene clusters 1, 3, and 4. Cluster 2 and 6 display a mixed pattern of increased accessibility and RNA expression, while cluster 5 genes, which are open and active in about 50% of wild-type cells, are less accessible and have decreased RNA levels in *Setdb1*-KO cells. (B) Gene expression intensity heatmap of aberrantly activated genes in Stem-I cell population from tamoxifen-treated Lgr5-GFP-Cre^ERT2^ (WT) and Lgr5-GFP-Cre^ERT2^/Setdb1^lox/lox^ (*Setdb1*-KO) mice. Panel at right shows the normalized intensity of the genes in other epithelial cell clusters from wild-type mice. (C) Heatmap with normalized motif deviation Z-scores of transcription factors in Stem-I cell population of tamoxifen-treated Lgr5-GFP-Cre^ERT2^ (WT) and Lgr5-GFP-Cre^ERT2^/Setdb1^lox/lox^ (*Setdb1*-KO) mice. Transcription factors whose binding motifs within the ATAC-seq peaks show significantly increased *Z* score values in Setdb1-deficient cells are listed. (D) Ridge plot of transcription factor motif accessibility z-scores across intestinal epithelial cell clusters. The density distribution of motif accessibility *Z* score for Nr1h3, Esrra, Atoh1, Nfya, Arid3a, Mafb, Bhlha15, and Phf21 is shown.

Indeed, single-cell Assay for Transposase-Accessible Chromatin sequencing (scATAC-seq) analyses identified a large number of new peaks and also a great number of peaks that were gained or lost in *Setdb1*-KO Stem-I cells compared to wild-type, control cells (Figures 5A and 5D). Peak-to-gene link analysis revealed consistently more genes with ATAC-seq peak link counts in *Setdb1*-KO cells compared to wild-type control (Figures 5B and 5C), implying that more open regulatory regions are connected to target genes as a result of *Setdb1* inactivation. Co-clustering analyses of the regions linked to genes in *Setdb1*-KO and wild type Stem-I cells (based on scATAC-seq and scRNA-seq signals), identified 6 major peak-to-gene clusters with significantly altered accessibility profiles and concomitant changes in gene expression (Figure 6A). Aberrantly accessible regions and linked activated genes in these clusters, included genes that are normally expressed in other differentiated intestinal epithelial cell types, such as the enterocyte-specific Rbp2, ApoC3, Papss2 genes, the Goblet cell-specific Tff3, Spink4, Mok genes, the Paneth cell specific Lyz1, Itln1 and several defensin genes, the Tuft cell specific Fes gene and a number of Entero-endocrine cell-specific genes, such as Gpx3, Itg9a, Pax4, Shc2, Slc8a1, Ambp (Figure 6B). These data point to a partial loss (cluster 5) and gain (especially cluster 1 and 3) of chromatin accessibility control in *Setdb1*-KO cells, which leads to aberrant and premature activation of differentiation stage-specific genes in Stem-I cells.

To gain mechanistic insights into how the above genes were activated prematurely in Stem-I cells, we performed TF motif enrichment analysis in the scATAC-seq regions detected in wild-type and *Setdb1*-KO Stem-I cells. We analyzed the enrichment of motifs beyond what is expected by chance, to reveal active binding by specific TFs at the accessible chromatin sites of interest. To correlate the motifs with the expression of the corresponding TF, which was inferred via gene activity scores, we calculated deviation Z-scores and identified the positively correlating TFs, demonstrating a direct link between TF abundance and regulatory activity in specific cell states (Figure 6C). We found that the *Setdb1* inactivation increased the motif accessibility and expression of 18 TFs in *Setdb1*-KO Stem-I cells, which are normally enriched in one or more differentiated cell types. As shown in the Ridge plots of Figure 6D, Atoh1, Mafk, and Bhlha15 TF motif exposure and expression are enriched in Goblet and EEC cells, Onecut2 motif in EEC cells, Ppara, Nr31c, and Esrra in enterocytes, and Arid3a in Tuft and EEC cells.

To validate the above findings, we performed regulon-based analysis for six of the above TFs using the PyScenic computational pipeline.^39^ PyScenic, examines coordinated expression of downstream target genes (regulons) and therefore evaluates TF activity from a complementary, transcriptional perspective, independent of chromatin accessibility data and thus provides an orthogonal assessment of TF function. Five out of six TF regulon scores were significantly enhanced in *Setdb1*-KO cells (Figure S6A), further supporting their role in premature gene activation.

**Figure 7:**
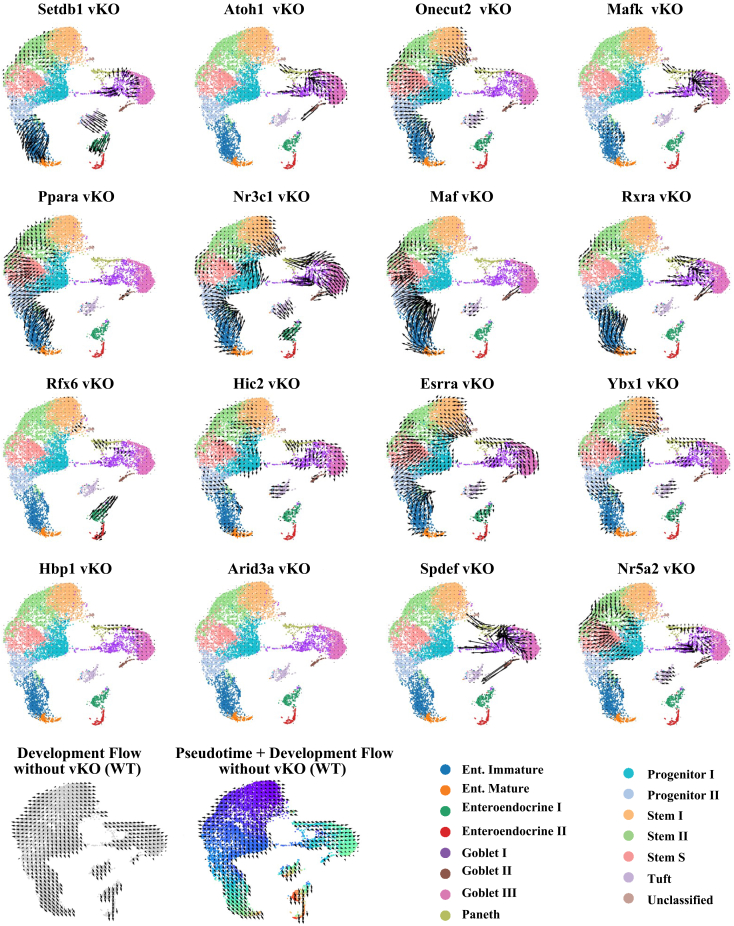
Virtual knockout of *Setdb1* and transcription factors with aberrantly exposed motifs in Stem-I cells displays a repertoire of divergent differentiation trajectories in the intestinal epithelium The panels show UMAP visualization of the trajectories with the simulation flow of in-silico perturbation experiment of Setdb1 (first panel) and the indicated transcription factors on the Lgr5-GFP-Cre^ERT2^ background computed by the CellOracle algorithm. The simulation flow (black arrows) demonstrates the loss of stability in the stem/progenitor compartment and the density and direction of the shift toward various lineage-committed states. Background is colored according to cell cluster identity. Bottom panels depict developmental flow vectors (left panel) and their overlay with cells, colored by pseudotime gradient from violet to red, indicating a normal differentiation trajectory in wild-type cells without virtual knock-out (panel at right).

The regulatory role of Setdb1 in proper intestinal stem cell differentiation was also examined by simulating the effect of its inactivation in silico, using the CellOracle,^40^ a machine-learning computational approach. We first constructed cluster-specific gene regulatory networks (GRNs) to identify key master TF regulators by integrating scRNA-seq and scATAC-seq data from wild-type Lgr5-GFP-Cre^ERT2^ cells. This information was then used for the simulation of cell-state transitions following single-gene perturbations by projecting results onto established cell trajectory maps. We performed virtual knockouts (vKO) of *Setdb1* and several lineage-specific regulators. The simulation flow for *Setdb1* vKO remarkably mirrored our *in vivo* experimental observations, i.e., a global loss of stability in the stem cell/progenitor pool characterized by disorganized, centrifugal vector fields pointing simultaneously toward multiple lineage outlets across the entire manifold (Figures 7 and S7).

To compare perturbation-induced flows with the endogenous developmental program, we calculated a developmental vector field from pseudotime following the CellOracle workflow. The similarity between each vKO perturbation vector and the developmental vector at the corresponding grid position was then quantified by the inner product (see STAR Methods). Positive inner-product values indicate local alignment between perturbation and developmental flow, whereas negative values indicate deviation from or opposition to the developmental direction. The magnitude of the inner product is influenced by both directional similarity and vector length.

In the case of *Setdb1* vKO, the inner-product scores were predominantly positive at intermediate pseudotime, indicating local alignment with developmental directions (Figure S8). However, because these positive signals emerged within a globally disordered and multi-directional perturbation field, they are best interpreted as evidence of aberrant multilineage priming and destabilized progenitor exit, rather than coordinated progression along the normal developmental trajectory. This interpretation is consistent with our *in vivo* observations showing impaired orderly differentiation, reduced enterocyte output, and the emergence of alternative differentiation trajectories upon Setdb1 loss.

In contrast to the broad effect of Setdb1 loss, perturbation of lineage-associated regulators, Atoh1 and Spdef,^41^^,^^42^^,^^43^^,^^44^ produced more spatially restricted and heterogeneous outcomes (Figure S8). Atoh1 expression is required for secretory lineage determination, and its loss of function results in the complete loss of secretory cells *in vivo*.^41^^,^^42^ Spdef functions downstream of ATOH1 in the secretory lineage. Loss of Spedf impairs the maturation of goblet and Paneth cells.^44^ In line with this, vKO of *Atoh1* and *Spdef* generated perturbation vectors concentrated within secretory domains, particularly around Goblet/Paneth-associated regions, and these were accompanied by predominantly negative or bidirectional inner-product signatures, indicating lineage-restricted deviation from the baseline developmental flow (Figures 7, S7, and S8).

By contrast, vKO of the other regulators, which are expressed in specific differentiated epithelial cell types, but are not considered lineage determinants, produced mixed patterns. vKO of the enterocyte-enriched *Ppara*, *Esrra*, and *Nr5a2* preferentially altered the absorptive axis, with mixed positive and negative inner-product values depending on pseudotime position, which is consistent with localized rewiring rather than global destabilization. vKO of the other TFs, including *Nr3c1*, *Rxra, Maf/Mafk, Onecut2, Rfx6*, and *Ybx1*, displayed distinct perturbation geometries, whereas vKO of factors such as *Arid3a* and *Hic2* had comparatively weak effects on the overall flow field (Figures 7, S7, and S8).

Collectively, the *in silico* results provide additional evidence that Setdb1 does not simply control a single differentiation path but rather functions as a global epigenetic gatekeeper that stabilizes transitional stem cell states and constrains unscheduled access to multiple lineage programs. Upon loss of Setdb1, cells are not uniformly redirected toward a single alternative fate. Instead, they undergo broad destabilization that permits simultaneous and inappropriate engagement of divergent transcriptional programs. This leads to disordered differentiation trajectories as evidenced by lineage-specific blockages or the activation of alternative differentiation paths in the *Setdb1*-KO mice.

**Figure 8:**
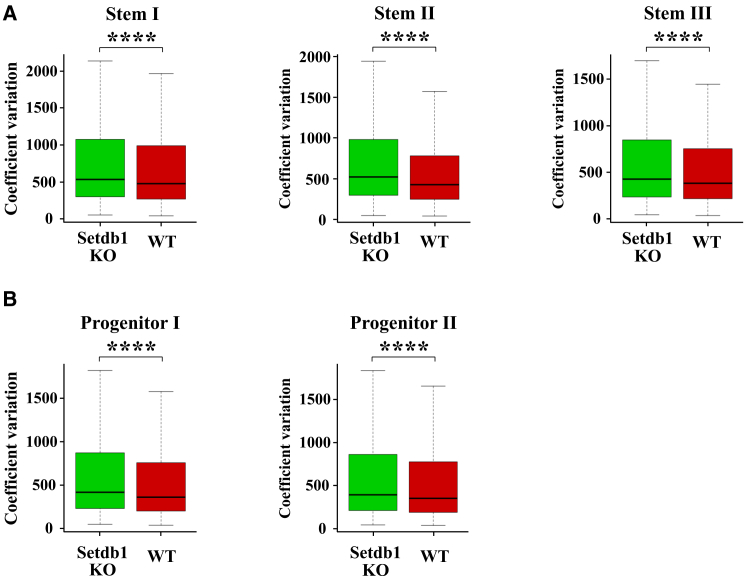
Increased cell-to-cell transcriptional variability in Setdb1-deficient Lgr5^+^ Stem and Progenitor cell clusters (A and B) Boxplots show coefficient variations per gene in cells within Stem-I, Stem-II, and Stem-III clusters (A) and within Progenitor-I and Progenitor-II clusters (B) from tamoxifen-treated Lgr5-GFP-Cre^ERT2^ (WT) and Lgr5-GFP-Cre^ERT2^/Setdb1^lox/lox^ (*Setdb1*-KO) mice. Mann-Whitney-Wilcoxon test, ∗∗∗∗*p* < 2.2 × 10^−16^.

The experimental and *in-silico* simulation results also suggest that premature exposure of TF binding sites in *Setdb1*-KO Stem-I cells is sufficient to promote stochastic binding of the TFs, even if they are expressed at low levels. This provides a mechanistic basis for the observed premature activation of differentiated cell type-specific genes, which could affect differential gene expression and trajectory establishment. Such changes may result in increased transcriptome variations. We therefore tested cell-to-cell variability of gene expression patterns in all stem cell and Progenitor cell clusters. Consistent with the increased global heterogeneity of the scATAC-seq peak areas in *Setdb1*-KO cells, we observed significant changes in Coefficient Variations per Genes in cells within individual stem cell and Progenitor cell clusters, indicating that *Setdb1* inactivation results in significantly enhanced cell-to-cell variations of cellular transcriptomes (Figures 8A and 8B).

### Moderate reduction of H3K9 methylation outside large heterochromatin domains correlates with the dynamic changes in the transposase-accessible chromatin areas in *Setdb1*-KO cells

**Figure 9:**
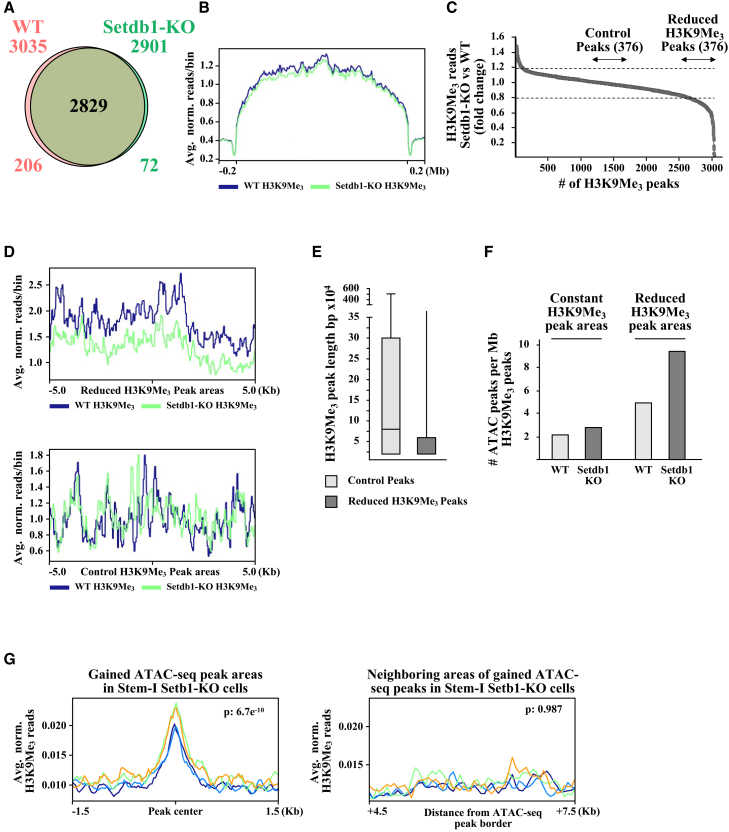
Differential effects of *Setdb1* inactivation on H3K9Me_3_-containing long heterochromatin domains and shorter peak areas in the vicinity of transposase accessible regions (A) Venn diagram shows the extent of overlap of H3K9Me_3_ CUT&Tag peaks between FACS-sorted GFP^+^ cells from tamoxifen-treated Lgr5-GFP-Cre^ERT2^ (WT) and Lgr5-GFP-Cre^ERT2^/Setdb1^lox/lox^ (*Setdb1*-KO) mice. The peaks were determined by the SICER broad ChIP-seq peak caller tool. (B) Average distribution profiles of the CUT&Tag reads within the SICER-called H3K9Me_3_ peaks indicated in (A). The graph shows average normalized reads per 50 bp bins. (C) Ranked coverage plots of the ratios of H3K9Me_3_ reads within the SICER-called H3K9Me_3_ peak areas between *Setdb1*-KO and WT cells. Dashed lines at ±20% are indicated as the base value of no significant change. The 376 peaks below the 0.8-fold cutoff are called “Reduced H3K9Me_3_ Peaks.” Control peaks are randomly selected peaks with *Setdb1*-KO/WT H3K9Me_3_ read ratios between 0.8 and 1.2-fold. (D) Average distribution profiles of H3K9Me_3_ reads within the 376 selected H3K9Me_3_ peaks containing a reduced number of reads in *Setdb1*-KO cells (upper panel) and the 376 randomly selected control H3K9Me_3_ peaks with unchanged H3K9Me_3_ reads (bottom panel), as indicated in (C). The graph shows average normalized reads per 50 bp bins. (E) Comparison of median peak lengths of the 376 control H3K9Me_3_ peaks and those with a reduced number of reads indicated in (C). (F) Evaluation of the overlaps between the peaks of the control and those with reduced H3K9Me_3_ reads in (C), with ATAC-seq peaks in cells from tamoxifen-treated Lgr5-GFP-Cre^ERT2^ (WT) and Lgr5-GFP-Cre^ERT2^/Setdb1^lox/lox^ (*Setdb1*-KO) mice. (G) Comparison of the average distribution of H3K9Me_3_ reads in the ATAC-seq peak areas gained in *Setdb1*-KO Stem-I cell cluster. Dark and light blue lines show H3K9Me_3_ reads in FACS-sorted GFP^+^ cells from two different tamoxifen-treated Lgr5-GFP-Cre^ERT2^/Setdb1^lox/lox^ (*Setdb1*-KO) mice. Green and orange lines show H3K9Me_3_ reads in GFP^+^ cells isolated from two different tamoxifen-treated Lgr5-GFP-Cre^ERT2^ (WT) mice. The graph shows average normalized reads per 50 bp bins. The statistical significance between the WT and *Setdb1*-KO profiles was determined by the Wilcoxon rank-sum test. *p* value for the ATAC-seq areas: 6.7e^−10^; *p*-value for the distant control areas: 0.987.

We compared the H3K9Me_3_ profiles of FACS-sorted control and *Setdb1*-KO Lgr5^+^-GFP cells. Consistent with the redundancy of Setdb1 function in generating H3K9 trimethylated nucleosomes,^5^^,^^7^^,^^9^^,^^14^ we did not observe significant differences in H3K9Me_3_ peaks between control and *Setdb1*-KO cells at the majority of large heterochromatin domains (Figures 9A, 9B, S6B, and S6C). However, comparing the H3K9Me_3_ CUT&Tag reads within the individual called peaks, we detected a substantial (>20%) drop in 376 out of the total 3035 peaks (Figures 9C and 9D). Importantly, the length of these 376 H3K9Me_3_ CUT&Tag peaks was significantly shorter and less variable, compared to the same number of randomly chosen peaks, in which H3K9Me_3_ reads were not affected by *Setdb1* inactivation (Figure 9E).

Next, we compared the locations of the existing and the new ATAC-seq peaks in the *Setdb1*-KO cells that overlap with H3K9Me_3_ peaks. As expected, the vast majority of the ATAC-seq peak areas, corresponding to accessible open chromatin, were excluded from the H3K9Me_3_ peaks marking closed heterochromatin domains (Figure S6B). Interestingly, however, the fraction of the ATAC-seq peaks that overlapped with the H3K9Me_3_ peaks was substantially enriched in the peak locations, with decreased H3K9Me_3_ read content in *Setdb1*-KO cells (Figure 9F). Next, we compared the H3K9Me_3_ status in the newly formed ATAC-seq peak areas in *Setdb1*-KO Stem-I cells, using the H3K9Me_3_ CUT&Tag datasets from GFP^+^ cells. Average coverage plots of H3K9Me_3_ reads from GFP^+^ cells centered on the newly formed ATAC-seq peak areas that were identified in *Setdb1*-KO cells revealed a small but statistically significant decrease of the H3K9Me_3_ signals in *Setdb1*-KO cells (Figure 9G), while in control areas outside the gained peak locations, H3K9Me_3_ levels were lower and not significantly altered in *Setdb1*-KO cells (Figure 9G).

Taken together, the above findings suggest that Setdb1-mediated H3K9 methylation regulates chromatin accessibility in a large number of gene-rich open genomic regions. In contrast, Setdb1 function in the majority of large heterochromatin regions is redundant.

## Discussion

Epigenetic enzymes regulate developmental programs through changes in chromatin structure, which influence the accessibility to transcriptional regulators.^7^^,^^11^ Regulation of chromatin accessibility involves dynamic events of heterochromatin compaction and structural transitions to open euchromatin, which control gene activity during ES cell differentiation to specific cell types or during developmental lineage specification.^9^^,^^10^^,^^11^^,^^12^^,^^13^

The main findings of this study demonstrate that the H3K9-methylase Setdb1, apart from its known function in the formation of large heterochromatin domains, plays an important role in controlling chromatin accessibility in open genomic regions in adult intestinal stem cells. This is demonstrated by the re-distribution of ATAC-seq peaks in *Setdb1*-KO cells, which mark transposase accessible open chromatin domains. Our results suggest that mechanisms involving localized changes within established chromatin compartments are important for fine-tuning gene expression patterns, in addition to the known regulatory role of heterochromatin to euchromatin transitions and vice versa. Such delicate regulatory mechanisms may prevent spurious activation or repression of genes, thereby contributing to adult stem cell maintenance and proper differentiation. In line with this notion, we found that the disruption of the tight control of chromatin accessibility in Lgr5^+^ intestinal stem cells leads to premature activation of genes whose expression is normally restricted to specific differentiated epithelial cell types. Premature gene activation is a consequence of premature exposure of TF binding sites in gene regulatory regions, due to the loss of Setdb1-dependent localized chromatin modifications.

The most apparent consequence of the resulting aberrant gene expression profiles is increased cell-to-cell transcriptional variability within well-defined cellular clusters. Cell-to-cell heterogeneity in gene expression, distinct from the intrinsic noise inherently present in cell populations, has been recognized as a potentially important feature to explain biological phenomena.^45^^,^^46^^,^^47^^,^^48^ For example, it has been shown that increased cell-to-cell transcriptional variability is coupled with the aging-dependent response of CD4^+^ T cells to immune stimulation,^49^ and in aging mouse muscle stem cells.^50^ Similar to the aging process, cellular differentiation is also tightly regulated by genetic and epigenetic factors and is modulated by stochastic signaling processes. Such multilevel control mechanisms generate temporally distinct transcriptomes, which characterize the identity of consecutive differentiation states. Since tight regulation is supposed to promote the creation of “more uniform” cellular transcriptomes, it is reasonable to assume that during the transition between the different cellular differentiation states, partial transcriptome diversification in individual cells may be part of the physiological differentiation process (schematically presented in Figure S9). Transition-specific variable transcriptomes must result from a stochastic equilibrium of regulatory factors and chromatin configurations, which determine the phenotypes of the previous and subsequent cellular states. Thus, we speculate that temporary variations restricted to the transition periods may represent a hallmark feature of cellular differentiation and lineage specification.

Disruption of the control mechanisms, such as those driven by the abnormal expansion of open chromatin domains in *Setdb1*-KO cells, can halt the progression of differentiation, resulting in an increased number of cells trapped at the transition states. In agreement with this scenario, we observed abnormal, premature activation of a number of differentiation stage-specific genes in *Setdb1*-KO Lgr5^+^ stem cells, which in turn elicit increased transcriptional variability in individual cells.

The consequences of abnormal gene activation may comprise alterations in stemness features and/or in the differentiation capacity of the cells. This possibility is supported by the strong dependence of Lgr5^+^ stem cells on Setdb1 function to generate intestinal organoid structures *in vitro*. The requirement of Setdb1 for the proper differentiation of intestinal stem cells was further demonstrated by the findings of *in vivo* lineage tracing assays, where a dramatic accumulation of stem cell clusters was observed in mice carrying conditionally inactivated *Setdb1* alleles. The number of mature absorptive enterocytes was severely reduced in *Setdb1*-KO Lgr5^+^ stem cell-derived progeny, in parallel with a comparable loss of cells in the Progenitor II cell compartment. According to our trajectory analysis, this latter cluster appears to represent a unipotent transition state toward the enterocyte lineage. This suggests that in the absence of Setdb1, the above differentiation path is blocked at the very early stage during the transition of Stem cells to Progenitor II cells.

Interestingly, all major secretory lineages were still readily identifiable among the progeny of Setdb1-deficient Lgr5^+^ stem cells, indicating that loss of Setdb1 does not block secretory differentiation per se. Only minor shifts were observed overall, with a slight reduction in Paneth cells and modest increases in Goblet, Tuft, and EEC cells. The one clear exception was the Goblet-I cell cluster, which expanded markedly, suggesting that this specific cell subtype is uniquely sensitive to Setdb1 loss. Goblet-I cell cluster appears to be derived directly from Progenitor I/TA cells and corresponds to the immediate precursor of Goblet-II and Goblet-III clusters. The accumulation of both Progenitor-I/TA and Goblet-I cell types following *Setdb1* inactivation suggests that cells escaping the first block of the hierarchical differentiation paths are halted at a second differentiation checkpoint at the Progenitor cell state. Nonetheless, the existence of Lgr5^+^ stem cell-derived secretory and endocrine-related cell types in *Setdb1*-KO mice suggests that these defects can be partially rescued by the activation of alternative lineage trajectories, through which they can arise directly from the Stem-I cell population. The activation of such alternative trajectories is likely to be facilitated by the increased transcriptional variability of cells that are blocked at intermediate differentiation checkpoints, which could provide cells with the flexibility to explore multiple differentiation directions (Figure S9).

Taken together, the results demonstrate that regulated chromatin accessibility impacts stage-specific transcriptome changes and plays an important role in executing proper differentiation paths in the intestinal epithelium. Perturbation of chromatin structure leads to aberrant and premature activation of genes. This results in increased cell-to-cell transcriptome variability for an extended period of time and in the inhibition of stem cell differentiation. Disruption of chromatin-controlled processes completely blocks differentiation into absorptive enterocytes. However, the activation of alternative differentiation paths compensates for the loss of secretory and endocrine-related cells, highlighting the remarkable plasticity in intestinal lineage specification regulation.

### Limitations of the study

The single-cell RNA-seq approach combined with single-cell ATAC-seq in this study provides an unprecedented resolution for identifying condition-dependent dynamics of individual cellular clusters within complex tissue and for understanding gene regulatory mechanisms involved in the transition between cellular states. However, single-cell approaches have some limitations, which should be acknowledged for the interpretation of the data of this study. First, despite the sophisticated statistical analyses employed, the intrinsic data sparsity and stochastic dropout phenomena characteristic of single-cell RNA sequencing may have precluded rare transcripts or transient changes in a small number of cells, which could result in missing regulatory pathways governed by TFs of low abundance. Second, the standard scRNA-seq approach used here does not provide spatial information, which would illuminate additional control mechanisms, especially in the intestinal epithelium, where the different cell types are hierarchically organized in the internal space of the gut. Third, the current study was designed to study *Setdb1* inactivation at a very early time point, before endogenous retrovirus activation, which would obscure specific chromatin structure-mediated effects of Setdb1 in the regulation of developmental genes. While the absence of substantial retrovirus activation was excluded by bulk RNA sequencing, one cannot exclude the possibility that it may have occurred in a small number of cells, which, due to the relatively low sequencing depth of the scRNA-seq approach, escaped detection. An additional limitation is coming from the fact that current single-cell ChIP-seq or CUT&Tag techniques lack robustness and cannot detect subtle but biologically relevant chromatin modification changes. For this reason, chromatin modification changes in Stem-I cell cluster were evaluated by bulk CUT&Tag assays in sorted GFP^+^ cells, which lowers detection limits when extrapolated to a specific subcluster of the respective cell population. Nevertheless, taking this into consideration, the small and statistically significant changes at the new ATAC-seq regions observed by bulk CUT&Tag point to a biologically relevant effect.

## Resource availability

### Lead contact

Further information and requests for resources and reagents should be directed to and will be fulfilled by the lead contact, Iannis Talianidis (talianid@imbb.forth.gr).

### Materials availability

Research materials generated in this study will be provided by the lead contact upon request.

### Data and code availability

• **Data:** Source Data files for the Total RNA-seq, scRNA-seq, scATAC-seq, and CUT&Tag sequence data have been deposited in the Gene Expression Omnibus (GEO) under accession number GSE279796 as listed in the key resources table.
• **Code:** All original code and computational analysis pipelines used in this study have been deposited at the web-based platform GitHub and are publicly available in https://github.com/GCMLab-Forth/scRNA_scATAC_analysis; https://github.com/Orsalia/Setdb_project.
• **Other items:** Any additional information required to reanalyze the data reported in this paper is available from the lead contact upon request.

## Acknowledgments

We thank M. Koukaki and E. Moltsanidou for technical assistance and discussions during the course of the work; E. Stratidaki and N. Gounalaki of the IMBB-Genomics facility and I. de la Rosa of the Core Genomics Unit of HMGU for assistance with the library preparations. This work was supported by the “Basic research Financing (Horizontal support of all Sciences)” under the National Recovery and Resilience Plan “Greece 2.0” funded by the European Union–NextGenerationEU, H.F.R.I. Project:15276 (to IT and M.D.L), the AXA Research Fund Chair in Epigenetics Program #2016 (to IT); the CIDEGENT program: CIDEXG/2023/30 and internal funds from the Helmholtz Pioneer Campus (to C.P.M-J).

## Author contributions

I.P. and I.T. conceived and designed the study. I.P., I.K.D., and H.K. performed experiments and evaluated the results. D.B., M.S., L.Z., I.G., and O.H. performed bioinformatics analyses of the data. E.D. analyzed histological data. C.P.M-J, M.D.L., and I.T. supervised the project. I.T. wrote the manuscript. All of the authors reviewed and edited the manuscript.

## Declaration of interests

The authors declare no competing interest.

## Declaration of generative AI and AI-assisted technologies in the writing process

No generative AI or AI-assisted technologies were used during the preparation of this work.

## STAR★Methods

### Key resources table

**Table:** 

REAGENT or RESOURCE	SOURCE	IDENTIFIER
**Antibodies**		

Goat anti-rabbit AlexaFluor 594	Cell Signaling	CAT#8889S RRID:AB_2716249
Goat anti-mouse AlexaFluor 488	Cell Signaling	CAT#4408S RRID:AB_10694704
Anti-HNF4a	Cell Signaling	CAT#3113 RRID:AB_2295208
Anti-Lyzozyme	ABCAM	CAT#AB36362 RRID:AB_776115
Anti-Mucin 2 (F2)	Santa Cruz Biotechnology	CAT#sc-515032; RRID: AB_2815005
Anti-H3K9Me3	ABCAM	CAT# AB8898 RRID:AB_306848

**Chemicals, peptides, and recombinant proteins**		

Advanced DMEM/F12	GIBCO	CAT#12634-010
N2 Supplement	GIBCO	CAT#17502048
B27 Supplement	GIBCO	CAT#12587010
N-acetylcysteine	SIGMA	CAT#A9165
EGF	R&D SYSTEMS	CAT#236-EG
Nicotinamide	SIGMA	CAT#N0636
Y27632	SIGMA	CAT#Y0503
SB431542	SIGMA	CAT#S4317
Fetal Bovine Serum (FBS)	GIBCO	CAT#10270-106
TrypLE	INVITROGEN	CAT#12604-013
Defined Trypsin Inhibitor	GIBCO	CAT#R007
Complete^TM^ EDTA-free protease Inhibitor Coctail	ROCHE	CAT#11836170001
CUTANA pA/G-Tn5	Epicypher	CAT# 23615-1017
TAPS	SIGMA	CAT#T9130
AMPure beads	Beckman Coulter	CAT#A63881
Optimal Cutting Temperature (OCT) medium	TISSUE-TEK	CAT#4583
DAPI	APPLICHEM	CAT#A4099
Mowiol® 4-88 Reagent	EMD Millipore	CAT#475904
G418	GIBCO	CAT#11811-031
Hygromycin	INVITROGEN	CAT#10687010
Matrigel	R&D SYSTEMS	CAT#3532-010-02
Concanavalin A-conjugated magnetic beads	Epicypher	CAT#23621-1401
Digitonin	APPLICHEM	CAT#A1905
Tamoxifen	MERCK	CAT#T5648

**Critical commercial assays**		

10x Genomics Single Cell 3’Reagent Kit v3.1	10xGenomics	CAT#1000268
Dual Index kit TT SetA	10xGenomics	CAT#1000215
Chromium Next GEM Single Cell ATAC Reagent Kit v1.1	10xGenomics	CAT#1000176
CollibriTM Library Quantification Kit	Thermo Fisher	CAT#A38524100
Bioanalyzer High Sensitivity DNA Analysis Assay Kit	Agilent	CAT#5067-4626
CORALL Total RNA Library preparation kit	Lexogen	CAT#175.24

**Deposited data**		

scRNA-seq, Total RNA-seq, scATAC-seq, CUT&Tag data	This paper	GSE279796

**Experimental models: Cell lines**		

L-WRN cells	ATCC	ATCC CRL-3276

**Experimental models: Organisms/strains**		

B6.129P2-*Lgr5*^*tm1(cre/ERT2)Cle*^/J	Barker at al.^25^ 2007; Jackson Laboratory	RRID: IMSR_JAX:008875
B6N.129S6-*Gt(ROSA)26Sor*^*tm1(CAG-tdTomato*^*∗*^*,-EGFP*^*∗*^*)Ees*^/J	Prigge et al.^51^ 2013; Jackson Laboratory	RRID: IMSR_JAX:023537
C57BL/6J	Jackson Laboratory	RRID: IMSR_JAX:000664

**Software and algorithms**		

CellRanger version 6.1.1	10xGenomics	https://github.com/10XGenomics/cellranger
DoubletFinder	McGinnis et al.^52^ 2019	https://github.com/chris-mcginnis-ucsf/DoubletFinder
Seurat	Butler et al.^53^ 2018	
SCP tool		https://github.com/zhanghao-njmu/SCP
Slingshot	Street et al.^35^ 2018	https://github.com/kstreet13/slingshot
ArchR	Granja et al.^54^ 2021	https://github.com/GreenleafLab/ArchR
FastQC		https://www.bioinformatics.babraham.ac.uk/projects/fastqc
TEtranscripts (version 2.2.3)	Jin et al.^55^ 2015	https://github.com/mhammell-laboratory/TEtranscripts
deepTools	Ramirez et al.^56^ 2016	https://github.com/deeptools/deepTools
MACS	Zhang et al.^57^ 2008	https://github.com/macs3-project/MACS
SICER2	Xu et al.^58^ 2014	https://github.com/zanglab/SICER2
Trimmomatic	Bolger et al.^59^ 2014	https://github.com/usadellab/Trimmomatic
HISAT	Kim et al.^60^ 2015	https://github.com/DaehwanKimLab/hisat2
Harmony version 4.1	Perkin Elmer	https://www.revvity.com/
Pyscenic	Van de Sande et al.^39^ 2020	
CellOracle	Kamimoto et al.^40^ 2023	https://github.com/morris-lab/CellOracle

### Experimental model and study participant details

#### Mice

Lgr5-GFP-Cre^ERT2^ (B6.129P2-*Lgr5*^*tm1(cre/ERT2)Cle*^/J)^25^ and ROSA(CAG-tdTomato∗,EGFP∗)Ees (called nTnG) (B6N.129S6-*Gt(ROSA)26Sor*^*tm1(CAG-tdTomato*^*∗*^*,-EGFP*^*∗*^*)Ees*^/J)^51^ mice were obtained from Jackson Laboratory. Setdb1^lox/lox^ mice carrying floxed exon 3 alleles were generated by crossing Setdb1^tm1a(EUCOMM)Wtsi^ mice, obtained from Wellcome Trust Sanger Institute, with B6.129S4-*Gt(ROSA)26Sor*^*tm2(FLP*^*∗*^*)Sor*^/J mice, obtained from Jackson Laboratory. After verifying flp recombinase-mediated deletion and recombination of the FRT site-flanked LacZ/neomycin cassette, the flp transgene was removed by serial back-crossing with C57Bl6 mice. Lgr5-GFP-Cre^ERT2^/ Setdb1^lox/lox^, Lgr5-GFP-Cre^ERT2^/nTnG and Lgr5-GFP-Cre^ERT2^/ Setdb1^lox/lox^/nTnG mice were obtained by crossing the respective mouse strains, as described in Figures S1B–S1D, respectively. The resulting mice were fertile and viable over 8 months of age. Mice were kept in grouped cages in a temperature-controlled, pathogen-free facility on a 12-hour light/dark cycle and fed with standard chow diet containing 19% protein and 5% fat (Altromin 1324) and water *ad libitum*. All animal experiments were approved by the Ethical Review Board of IMBB-FORTH and the Animal Ethics Committee of the Prefecture of Crete (#90835) and were performed in accordance with the respective national and European Union regulations. All experiments were performed in randomly chosen age-matched male mice. No blinding was used in this study. Unless otherwise indicated, mice were treated and analyzed at 60-65 days after birth. Treatment of mice were performed with intraperitoneal injections of either vehicle (corn-oil) or 70 mg/kg Tamoxifen.

### Method details

#### Isolation of intestinal epithelial cells

To isolate crypts, the ileum was dissected and washed with cold phosphate buffered saline (PBS) using blunt-ended syringe, opened longitudinally with a scissor and cut into 3-5 mm pieces. After extensive washing with cold PBS, the tissue fragments were resuspended in 30 volumes of cold PBS, containing 20 mM EDTA and incubated with constant shaking for 30 min at 4°C. The tissue fragments allowed to settle by gravity to remove supernatant and resuspended in cold PBS. After vigorous shaking and trituration with 10ml pipette the tissue pieces were collected by gravity sedimentation. The supernatant was discarded and the PBS extraction was repeated 7 to 9 times, until the supernatant contained mainly crypts, as verified by microscopic examination. The final supernatant containing enriched crypt fraction was passed through 70 μm cell strainer and centrifuged at 400g for 5 min. To isolate epithelial cells, the crypt pellets were incubated in 10 volumes of preheated TrypLE Express (Gibco) for 4.5 min at 37°C. The reactions were stopped by the addition of equal volume of Defined Trypsin Inhibitor (Gibco). The cells were passed through 40 μm cell strainer, centrifuged at 400g for 5 min and resuspended in ice-cold PBS containing 1 mM EDTA and 2% FBS. GFP^+^ cells were sorted using BD FACSAria III cell sorter gated by forward scatter, side scatter and pulse width parameter at a flow rate of 3-5000 events/second.

#### Immunostaining assays

For immunofluorescence staining, freshly isolated ileal fragments were cut longitudinally and washed with PBS. The tissue fragments were prefixed in 4% paraformaldehyde (PFA) in PBS for 2 hours at room temperature and treated 30% ice cold sucrose, before embedding in Optimal Cutting Temperature (OCT) embedding medium and were frozen in liquid nitrogen. Frozen sections (5 to 7 μm thick) were air dried blocked in 5% BSA in PBS containing 0.1% Triton X-100 for 1 hour and then incubated with 1% BSA, 0.1% Triton X-100 in PBS with the indicated primary antibodies at 4°C overnight. After incubation with AlexaFluor 594- or AlexaFluor 488-conjugated goat anti-rabbit or anti-mouse secondary antibodies for 1 hour at room temperature and counterstained with 1 μg/ml DAPI for 10 min, the slides were covered with Mowiol® 4-88 Reagent (EMD Millipore, 475904), as described.^61^ Fluorescence images were observed using a Leica TCS SP8 confocal microscope.

#### High content microscopy analyses of wide field and confocal images

Isolated intestinal epithelial cells were plated onto glass cover slips and fixed with 4% paraformaldehyde (PFA). Following antibody staining, immunofluorescence images were acquired with a 20x or 40x lens (Olympus, Shinjuku City, Tokyo, Japan) with an Operetta High Content Screening Microscope (HCSM, PerkinElmer, Waltham, MA, USA) in wide field and analyzed using Harmony software v4.1.

To estimate relative protein expression levels, antibody-stained epithelial cells were segmented based on DAPI nuclear staining. Clusters of adjacent cells were excluded from the analysis. Among the remaining cells, only GFP-positive populations were selected, and for these, the nuclear sum intensity of the target protein signal was quantified. The resulting values were plotted using GraphPad Prism v6.

#### Organoid cultures

About 500 FACS-sorted GFP^+^ cells were resuspended in 50 μl Matrigel and applied to pre-warmed tissue culture plates to form hemispherical droplets. Following solidification, medium containing Advanced DMEM/F12, 2 mM Glutamax, 10 mM Hepes, 100 U/ml penicillin, 100 μg/ml streptomycin, N2 Supplement (1x), B27 Supplement (1x), 1 mM N-acetylcysteine, 50 ng/ml EGF, 10 mM Nicotinamide, 10 μM Y27632, 10 μM SB431542 and 50% conditioned WRN medium was added to cover the Matrigel domes. The medium was changed every 2 days for the indicated times.

Conditioned WRN medium was prepared from L-WRN (ATCC CRL-3276) cells expressing Wnt3A, R-spondin and Noggin.^62^ The cells were grown in DMEM/10%FBS supplemented with 0.5 mg/ml G418 and 0.5 mg/ml Hygromycin. To prepare conditioned medium, the cells were split and grown in DMEM/10%FBS, without the selection antibiotics until confluence. Fresh DMEM was added without FBS and the cells were incubated for 24 hours at 37°C. The supernatant was collected and the plates were replaced with new medium. Supernatants from 4 successive 24-hours incubations were collected and stored in -20°C. For passaging organoids, the cells in Matrigel were resuspended in 500 μl TrypLE Express supplemented with 10 μM Y-27632 and incubated for 5 min at 37°C. After the addition of equal volume of Defined Trypsin Inhibitor, the suspension was extensively triturated using glass Pasteur pipette. After counting the cells were re-seeded using Matrigel.

#### CUT&Tag assays

CUT&Tag assays were performed as described previously.^63^ Briefly, nuclei were prepared from the isolated, FACS-sorted GFP^+^ intestinal epithelial cells by resuspension in 10 volumes of ice-cold Swelling Buffer, containing 10 mM Tris pH 7.5, 2 mM MgCl_2_, 3 mM CaCl_2_ and protease inhibitor cocktail (Complete^TM^ EDTA-free, Roche). After centrifugation at 400g for 5 min at 4°C, the pellet was resuspended in Lysis Buffer, containing 10 mM Tris pH 7.5, 2 mM MgCl_2_, 3 mM CaCl_2_, 10% glycerol, 1% NP40 and protease inhibitor cocktail. After incubation in ice for 3 min the nuclei were collected by centrifugation for 5 min at 400g, at 4°C, washed twice with PBS. Then, 10^5^ nuclei were resuspended in a buffer containing 20 mM HEPES–KOH pH 7.9, 10 mM KCl, 0.1% Triton X-100, 20% Glycerol, 0.5 mM Spermidine, protease inhibitor cocktail and incubated for 10 min with Concanavalin A-conjugated magnetic beads (Epicypher) that were previously activated by repeated washings with a buffer containing 20 mM HEPES pH 7.9, 10 mM KCl, 1 mM CaCl_2_, 1 mM MnCl_2_. The reactions were supplemented with 0.5 volume of a buffer containing 20 mM HEPES-NaOH, pH 7.5, 150 mM NaCl, 0.5 mM Spermidine, protease inhibitor cocktail, 0.01% Digitonin, 2 mM EDTA and 0.5 μg of the anti-H3K9Me_3_ or IgG negative control primary antibodies. The samples were incubated overnight at 4°C by gentle agitation. The beads were then supplemented with a buffer containing 20 mM HEPES-NaOH, pH 7.5, 150 mM NaCl, 0.5 mM Spermidine, protease inhibitor cocktail, 0.01% Digitonin and 0.5 μg of anti-rabbit secondary antibody (Epicypher). After incubation at room temperature for 30 min, the beads were washed twice with the same buffer and resuspended in the same buffer containing 300 mM NaCl. Protein A/G-fused Tn5 transposase protein (CUTANA pA/G-Tn5, Epicypher) was added and the samples were incubated for 1 hour at room temperature followed by washing with a buffer containing 20 mM HEPES-NaOH, pH 7.5, 300 mM NaCl, 0.5 mM Spermidine, protease inhibitor cocktail and 0.01% Digitonin. The beads were collected and resuspended in the above buffer containing 10 mM MgCl_2_, incubated for 1 hour at room temperature, washed once with 10 mM TAPS pH 8.5 and 0.2 mM EDTA and incubated with 10 mM TAPS pH 8.5 and 0.1% SDS for 1 hour at 58°C, to release DNA. After quenching by the addition of 3 volumes of 0.67% Triton-X100, the samples were subjected to PCR reaction (14 cycles) using universal i5 primers and barcoded i7 primers. After cleanup with AMPure beads (Beckman Coulter), quantification and quality evaluation in Agilent Bionalyzer, the libraries were sequenced using Illumina NextSeq500 platform.

#### Single-cell RNA-seq sample preparation and sequencing

After isolation of GFP^+^ cells using BD FACSAria III cell sorter, viable cells were fixed using 10% DMSO in media and stored at -80°C until further use. Two independent biological replicates were used for Lgr5^+^ WT and Lgr5^+^ *Setdb1-*KO mice respectively. Cells were washed twice in PBS/ BSA 0,02% and centrifuged at 300 × g for 5 min at 4°C. The samples were processed according to the 10x Genomics Single Cell 3′ Reagent Kit User Guide. cDNA was assessed using a Bioanalyzer High Sensitivity DNA Analysis assay (Agilent) and the libraries were quantified using the Collibri™ Library Quantification Kit (Thermo Fischer Scientific, A38524500) in a QuantStudio™ 6 Flex Real-Time PCR System (Thermo Fisher Scientific). The pooled library was sequenced using SP100 flow cells in a NovaSeq6000 sequencer (Illumina) at the HMGU Core Facility for NGS Sequencing at a sequencing depth of 40,000 reads per cell.

#### Single-cell ATAC-seq library preparation and sequencing

A single-cell paired-matched analysis of the assay for transposase-accessible chromatin with sequencing (scATAC-seq) was performed with approximately 300,000 pre-sorted cells from the same cell suspension that were used for scRNAseq library preparation. The cells were pelleted by centrifugation at 300 × g for 5 min at 4°C, washed twice with pre-chilled 1 ml of PBS/0.2%BSA and resuspended in 100 ml of Lysis buffer for 5 minutes, according to the Nuclei Isolation for Single Cell ATAC Sequencing demonstrated protocol (CG000169, 10X Genomics). The isolated nuclei were counted under a microscope and proceeded to Tagmentation reaction according to the manufacturer’s protocol (Chromium Next GEM Single Cell ATAC Reagent Kits v1.1, manual CG000209, RevD). Briefly the nuclei after transposition were loaded in a chip H and after GEM generation and clean-up, they were subjected to 9 cycles indexing PCR reaction. The final scATAC libraries were assessed using a Bioanalyzer High Sensitivity DNA Analysis assay (Agilent) and the Collibri™ Library Quantification Kit as above. Sequencing was performed in a SP 100 flow cell in a NovaSeq 6000 sequencer (Illumina) at the HMGU Core Facility for NGS Sequencing with the sequencing length recommended by 10x Genomics (50, 8, 16, 50). On average, 50,000 reads per nucleus were yielded.

#### Single-cell RNA-seq data analysis

Initial preprocessing was performed using the CellRanger pipeline (version 6.1.1, 10x Genomics), including alignment to the reference genome, barcode demultiplexing, and unique molecular identifier (UMI) counting. The CellRanger web summary report provided metrics such as the estimated number of cells, median genes per cell, and the percentage of reads mapping to the genome.^64^

To identify and remove potential doublets, the DoubletFinder algorithm^52^ was applied using the following parameters: pN = 0.25, pK = 0.09, the expected number of doublets (nExp), and the first 10 principal components (PCs = 1:10). The doublet detection rate was based on nExp, calculated from the estimated number of cells and the experimental protocol. Cells identified as doublets were excluded from further analysis.

Post-doublet removal, quality control filtering was performed using the Seurat package (version 5.0.3)^57^ to retain high-quality cells. Cells were filtered based on the following criteria: a number of detected features (genes) per cell between 200 and 4000, a mitochondrial gene expression percentage below 5%, and a total RNA count not exceeding 18,000.

Normalization was conducted using the “LogNormalize” method, followed by the identification of the top 2,000 highly variable genes with the variance-stabilizing transformation (vst) method. The data were then centered and scaled, and principal component analysis (PCA) was used for dimensionality reduction. A nearest neighbor graph was constructed using the top 10 principal components, and cells were clustered using the Louvain algorithm at a resolution of 0.5 via the Seurat FindClusters function. Clustering was visualized with Uniform Manifold Approximation and Projection (UMAP). The resulting Seurat object was then input into the SCP (Single-Cell Pipeline) tool (https://github.com/zhanghao-njmu/SCP) for visualization and trajectory inference using the Slingshot algorithm.^35^

#### Assessment of cell-to-cell transcriptome variation

Cell-to-cell heterogeneity in mRNA expression was assessed as described before^49^ by calculating the coefficient of variation (CV) in gene expression separately for wild-type and knockout cells. Gene count matrices were generated for each condition, and the CV for each gene was computed as the standard deviation of gene expression divided by the mean expression, multiplied by 100. Boxplots were generated to visualize differences in CV between conditions, and statistical significance was assessed using a Wilcoxon rank-sum test.

#### Single-cell ATAC-seq data analysis

Initial preprocessing was performed using the CellRanger pipeline (version 6.1.1, 10x Genomics), including alignment of reads to the reference genome and generation of BAM files. The BAM files were normalized using the *bamCoverage* tool from deepTools (version 3.3.2)^59^ with the BPM (Bins Per Million) method. We filtered out cells with less than 2.000 fragments and cells with TSS enrichment less than 4. For dimensionality reduction, Latent Semantic Indexing (LSI) was employed using the *addIterativeLSI* function of ArchR.^54^ Cells were clustered based on the reduced LSI space using a k-nearest neighbors (kNN) algorithm (*addClusters* function). The resolution of clustering was determined empirically to maximize biological relevance. Gene scores were calculated with the *addGeneScoreMatrix* function, which estimates gene expression by computing accessibility of regulatory elements near gene bodies and promoters. Integration of scATAC-seq and scRNA-seq data was carried out using ArchR’s *addGeneIntegrationMatrix* function. Gene expression profiles from matched scRNA-seq data were mapped to scATAC-seq cells by comparing the gene score matrix from scATAC-seq with the gene expression matrix from scRNA-seq. Cells were paired based on similarity in their profiles, allowing the assignment of gene expression information to the scATAC-seq cells.

Peak calling of scATAC-seq was performed as follows. After the integration was made, we performed peak calling per cluster per condition. Using the functions of *addGroupCoverages* and *addReproduciblePeakSet* ArchR first builds a coverage profile for each group of cells and then calls Peaks using MACS2.^57^ Then, with the *getBW* function and *ReadsInTSS* normalization method bigwig files were generated that were used for the cluster specific browser plots and the intensity heatmaps (Figure 6A). Intensity plots of ATAC-seq signals were generated using the *computeMatrix* function of *deeptools* with reference points. To create the reference peak list, we used the union of common and unique peaks of Stem-I-WT and Stem-I-KO cells obtained by the function intersect and subtract of bedtools (Figure 6A).

#### Peak-to-gene linkage analysis

To explore potential regulatory relationships between chromatin accessibility and gene expression, peak-to-gene linkage analysis was performed using the *addPeak2GeneLinks* function in ArchR^54^ with a maximum distance of 250.000bp, a correlation cutoff equal to 0.45 for only the Stem-I-KO and Stem-I-WT cells. This analysis identifies correlations between peaks and gene expression by considering accessibility and expression values across cells. The fact that two elements are only accessible in a particular cell community does not necessarily mean that they have a regulatory relationship with each other. However, when a reachable peak is continuously accessible whilst a nearby gene is transcribed, we can deduce that this peak is a potential regulatory transcriptional element of the associated (linked) gene. Finally, results were visualized with the *plotPeak2GeneHeatmap* function, which displays the peak-to-gene links for cells within the Stem-I cluster using an FDR cutoff of 1e^-04^. When we used the *addPeak2GeneLinks* function for only the Stem-I-WT and then only for the Stem-I-KO we could generate the number of peaks that were associated for each gene in these two conditions. Cumulative distribution analysis (R) allows to represent the proportion of regions with more or less than a specific threshold value for peak-to-genes observations (number of ATAC regions associated with one given gene).

#### Motif analysis and identification of transcriptional regulators

Motif annotations of our scATAC-seq regions were added using the CIS-BP motif database (*addMotifAnnotations*, and background peak sets were generated with *addBgdPeaks* to enable bias-corrected analyses. ChromVAR deviation scores were calculated with *addDeviationsMatrix* to quantify motif accessibility deviations across groups. Correlation analyses between motif accessibility (MotifMatrix) and gene expression (GeneIntegrationMatrix) were performed with *correlateMatrices*. Motifs were classified as active transcription factors if they displayed strong positive correlation with gene expression (correlation > 0.5), statistical significance (p adjusted < 0.05). Motif activity z-scores of active Transcription Factors were aggregated by cell type and condition using getGroupSE and were plotted normalized (Figure 6C) and as a distribution (Figure 5D).

#### Analysis of transcription factor regulons

Regulatory Networks for selected Transcription Factors were identified using PyScenic pipelines,^39^ using as potential Transcription factors the *mm_mgi_tfs.txt* list provided by the Pyscenic tutorial pipelines and as motive database: *mm10_10kbp_up_10kbp_down_full_tx_v10_clust.genes_vs_ motifs.rankings.feather.* To ensure reproducibility we run the pipelines 10 times with a different starting seed before processing the results. For the resulting regulon files, we kept as “significant” regulon the regulons with the maximum NES score (i.e., those with the best motive enrichment score). For all the appearing regulons, we summed all the AUC scores per condition and divided them by 10.

Those mean AUC scores were integrated to the *scanpy* object. To measure the importance of the difference of regulons activity score between clusters, we performed either t-test of Mann-Whitney test, based on the existence or not of the normality of the cluster AUC scores. We then corrected for multiple testing and used the p-adjusted values for our next comparisons. Our null hypothesis was that the Knockout cluster presented less activity on these regulons.

#### Analysis of H3K9me_3_ CUT&Tag data

For the analysis of H3K9me_3_ CUT&Tag data, the quality of all FASTQ files was assessed using FastQC (https://www.bioinformatics.babraham.ac.uk/projects/fastqc). Low-quality bases and adapter sequences were trimmed using Trimmomatic (version 0.39).^59^ The cleaned FASTQ files were aligned to the UCSC mm10 mouse genome using HISAT2 (version 2.1.0)^63^ with default settings, retaining only reads with a mapping quality score greater than 30. BAM files were converted to BigWig format using deepTools (version 3.3.2)^59^ and visualized in the UCSC Genome Browser. Read counts were standardized by down sampling to match the sample with the fewest reads, and peak regions were identified using SICER2.^58^

Average coverage profiles across peak centers of H3K9me_3_ and ATAC-seq data were computed and visualized using DeepTools.^56^ H3K9Me3 chip-seq aligned bam files for the wild-type and *Setdb1*-KO were normalized according to their geometric mean. The sum of reads from each of the 4 individual samples was estimated and their geometric mean across samples was evaluated. Dividing each sum by the geometric mean resulted in the scaling factors and the reads from each sample library were divided by this factor. The normalized libraries were converted to bigwigs using genomeCoverage bedtools function. The computeMatrix function was used as follows : *computeMatrix reference-point --referencePoint gene -b 1500 -a 1500 -R genes.bed -S sampleKO1.bw sampleKO2.bw sample1.bw sample2.bw --shift 0 0 30 30 --binSize 10 -o genes_shifted_matrix.gz.* Plotprofile function was then evaluated, *i.e plotProfile∖-m genes_shifted_matrix.gz—samplesLabel “sample12”KO “sample2_shift+150_WT” ∖ -out genes_two_profiles_shifted.png*.

#### Total RNA purification and RNA-sequencing for retroviral transcript detection

Total RNAs were purified from the FACS-sorted GFP^+^ epithelial cells using Trizol extraction, as described previously.^61^ Briefly, the cells were resuspended in 10 volumes of Trizol reagent by brief vortexing, followed by incubation at room temperature for 5 min. After the addition of 0.2 volumes of chloroform and further incubation at room temperature for 3 minutes, the samples were centrifuged at 12000 g for 15 minutes at 4°C and the aqueous phase was collected and precipitated by the addition of equal volume of isopropanol. After 10 minutes’ incubation at room temperature, the RNA was collected by centrifugation at 12000g for 15 minutes. The pellet was resuspended in H_2_O and re-precipitated with ethanol. The RNA samples were further purified by digestion with 10 units of DNase-I for 10 min at 37°C, followed by purification with phenol/chloroform extraction and ethanol precipitation.

To obtain maximal 5’ coverage, RNA-seq libraries were generated using CORALL Total RNA Library preparation kit from Lexogen and sequenced in an Illumina NextSeq 500 system.

Transposable element (TE) and gene expression levels were quantified using TEtranscripts (version 2.2.3)^65^. RNA-seq data were aligned to the reference genome, and TEtranscripts was used to estimate the expression of both TEs and protein coding genes. Following the differential expression analysis, RPKM (Reads Per Kilobase of transcript, per Million mapped reads) values were calculated for comparisons of individual TE transcripts and protein coding transcripts.

#### Data processing for trajectory inference and gene regulatory network (GRN) construction

The scRNA-seq datasets for control (Lgr5-GFP-Cre^ERT2^) and *Setdb1*-KO (Lgr5-GFP-Cre^ERT2^/Setdb1^lox/lox^) were processed using the Seurat (v4.0) and Scanpy (v1.9) frameworks. Low-quality cells with fewer than 200 genes or mitochondrial content exceeding 10% were excluded. After normalization and principal component analysis (PCA), dimensionality reduction was performed using UMAP to visualize cell clusters and lineages. Developmental trajectories and lineage branching were inferred using Slingshot, with Lgr5^+^ Stem-I cell clusters designated as the starting point. For temporal resolution, Diffusion Pseudotime (DPT) was calculated to map the progression of cells from a stem-like state to terminal differentiated fates across the intestinal manifold.

To model the regulatory landscape, we employed CellOracle (v0.12.0) to reconstruct gene regulatory networks (GRNs).^40^ A base GRN was first established using the CellOracle built-in database to link transcription factors (TFs) to their potential target genes. We then refined this network using our control (Lgr5-GFP-Cre^ERT2^) scRNA-seq data to build a cluster-specific GRN, utilizing the *oracle.get*_cluster_specific_Trancription Factor network function to identify active regulatory links within the intestinal epithelium.

#### In silico gene perturbation and virtual knockout (vKO) analysis

To evaluate the functional impact of Setdb1 and its downstream targets, we performed in silico gene perturbation analysis using the CellOracle framework.^40^ First, *Setdb1* was virtually inactivated by setting its expression to zero in the control (Lgr5-GFP-Cre^ERT2^) gene regulatory network (GRN) model. The resulting shift in cell state was computed using *oracle.simulate_shift*, and the predicted perturbation vectors were projected onto the UMAP manifold as a vector field. We then perturbed 15 transcription factors identified as aberrantly activated or associated with increased chromatin accessibility in Setdb1-deficient cells and examined the direction and magnitude of the predicted vector flows for each virtual knockout (vKO).

To compare perturbation-induced flows with the endogenous developmental program, we additionally calculated a developmental vector field from pseudotime following the CellOracle workflow. Briefly, pseudotime values were transferred onto a digitized two-dimensional grid defined on the embedding, and the spatial gradient of pseudotime was computed to generate a baseline developmental field. The similarity between each vKO perturbation vector and the developmental vector at the corresponding grid position was then quantified by the inner product (dot product):

$${\text{IP}}_{i}=v_{i}^{\;\text{pert}}\cdot v_{i}^{\;\text{dev}}\text{,}$$

where $v_{i}^{\;\text{pert}}$ denotes the CellOracle-predicted perturbation vector and $v_{i}^{\;\text{dev}}$ the pseudotime-derived developmental vector at grid point *i*. Positive inner-product values indicate local alignment between perturbation and developmental flow, whereas negative values indicate deviation from or opposition to the developmental direction; the magnitude of the inner product is influenced by both directional similarity and vector length.

### Quantification and statistical analysis

Comparisons between two groups were performed using Mann-Whitney U-test or Mann-Whitney-Wilcoxon test or Student’s t-test, as indicated in the legends. scRNA-seq, scATAC-seq, CUT&Tag data were obtained from at least two biological replicates.
