# Supplementary material for: Setdb1 safeguards proper differentiation of adult intestinal stem cells by controlling chromatin accessibility and transcriptome variability
Source: iScience. 2026 Jul 9;29(8):116731. doi: 10.1016/j.isci.2026.116731 (PMC13380120; doi:10.1016/j.isci.2026.116731)
Supplement: Document S1. Figures S1–S9 [file mmc1.pdf]

iScience, Volume *29*

Supplemental information

Setdb1 safeguards proper differentiation of adult intestinal stem cells by controlling chromatin accessibility and transcriptome variability

Ioanna Peraki, Liwei Zhang, Dimitris Botskaris, Marianna Stagaki, Ioannis K. Deligiannis, Haroula Kontaki, Elena Deligianni, Ioannis Giannoulakis, Orsalia Hazapis, Matthieu D. Lavigne, Celia P. Martinez-Jimenez, and Iannis Talianidis

Figure S1

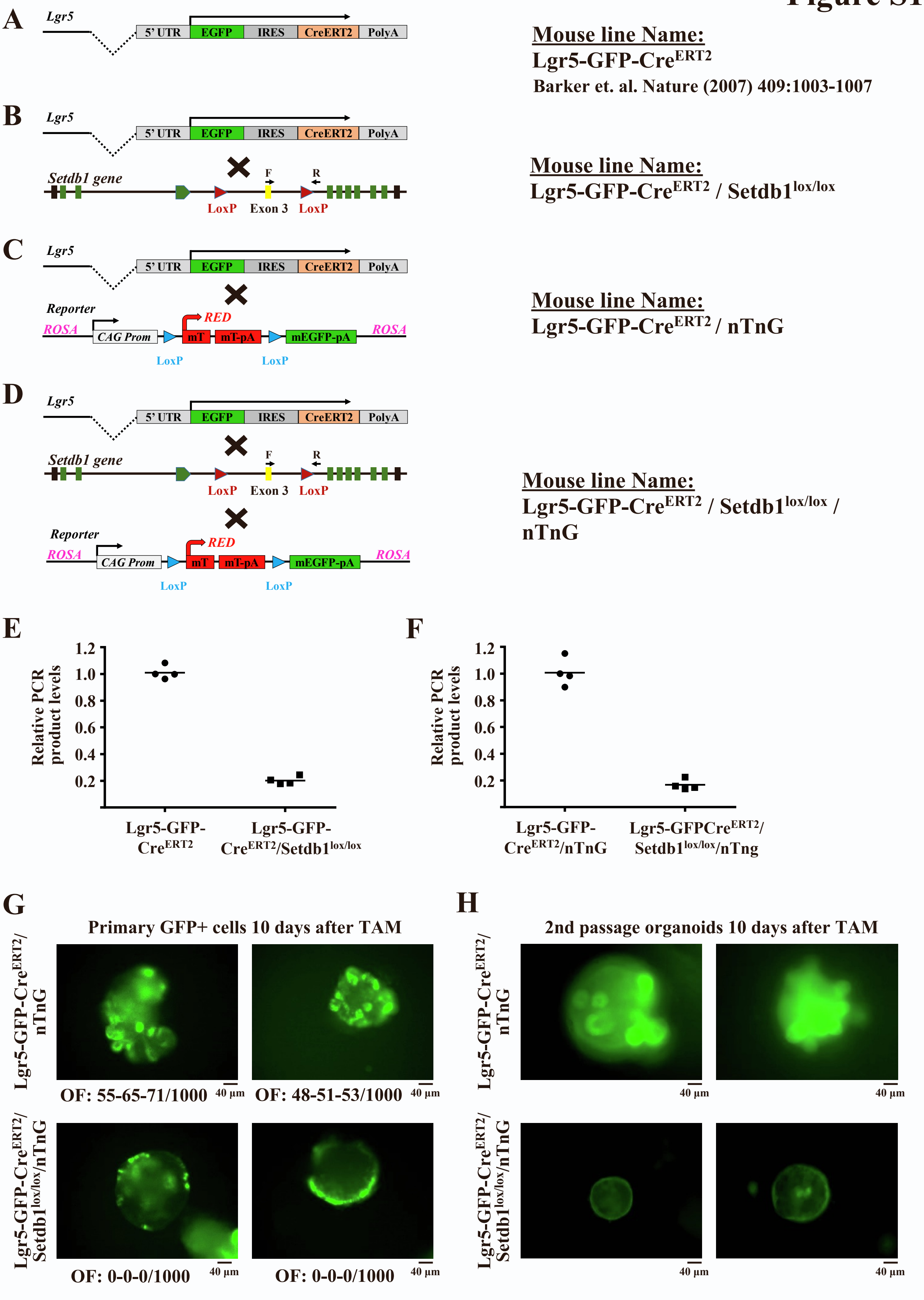
A
Lgr5
5' UTR
EGFP
IRES
CreERT2
PolyA
Mouse line Name:
Lgr5-GFP-CreERT2
Barker et. al. Nature (2007) 409:1003-1007
B
Setdb1 gene
F
R
LoxP
Exon 3
LoxP
Mouse line Name:
Lgr5-GFP-CreERT2 / Setdb1lox/lox
C
Reporter
ROSA
CAG Prom
RED
mT
mT-pA
mEGFP-pA
LoxP
Mouse line Name:
Lgr5-GFP-CreERT2 / nTnG
D
Mouse line Name:
Lgr5-GFP-CreERT2 / Setdb1lox/lox / nTnG
E
Relative PCR product levels
1.2
1.0
0.8
0.6
0.4
0.2
Lgr5-GFP-CreERT2
Lgr5-GFP-CreERT2/Setdb1lox/lox
F
Lgr5-GFP-CreERT2/nTnG
Lgr5-GFPCreERT2/Setdb1lox/lox/nTng
G
Primary GFP+ cells 10 days after TAM
Lgr5-GFP-CreERT2/nTnG
Lgr5-GFP-CreERT2/Setdb1lox/lox/nTnG
OF: 55-65-71/1000
OF: 48-51-53/1000
OF: 0-0-0/1000
OF: 0-0-0/1000
40 µm
H
2nd passage organoids 10 days after TAM

Figure S1

Schematic presentation of the genotypes of mouse models, analysis of Setdb1-exon 3 deletion and quantitation of immune-stained HNF4a^{+}, Lysozyme^{+} and Mucin^{+} cells in wild-type and Setdb1-deficient ileal epithelium. Related to Figure 1 and Star Methods.

(A) Lgr5-GFP-Cre^{ERT2} mice described in Ref 22, contains one knock-in allele expressing GFP and Cre^{ERT2} specifically in Lgr5^{+} stem cells.

(B) Crossing Lgr5-GFP-Cre^{ERT2} mice with Setdb1$^{lox/lox}$ mice generates Lgr5-GFP-Cre^{ERT2}/ Setdb1$^{lox/lox}$ mice, which upon tamoxifen treatment results in the deletion of exon 3 of Setdb1 and the generation of a premature stop codon in the area of exon 4, specifically in Lgr5^{+} stem cells.

(C) Crossing Lgr5-GFP-Cre^{ERT2} mice with ROSA(CAG-tdTomato*EGFP*)Ees mice generates mice named Lgr5-GFP-Cre^{ERT2}/nTnG, which express tomato-red in all cells and GFP and Cre^{ERT2} specifically in Lgr5^{+} stem cells. Upon tamoxifen treatment, all Lgr5^{+} stem cell progeny will express GFP from both Lgr5 and the ROSA locus. In parallel, these cells also loose tomato-red expression.

(D) Crossing Lgr5-GFP-Cre^{ERT2} mice with Setdb1$^{lox/lox}$ and ROSA(CAG-tdTomato*EGFP*)Ees mice, generates mice named Lgr5-GFP-Cre^{ERT2}/ Setdb1$^{lox/lox}$/nTnG. These mice express tomato-red in all cells and GFP and Cre^{ERT2} specifically in Lgr5^{+} stem cells. Upon tamoxifen treatment, exon 3 of Setdb1 is deleted specifically in Lgr5^{+} stem cells and all Setdb1-deficient Lgr5^{+} stem cell progeny will express GFP from both Lgr5 and the ROSA locus.

(E-F) Deletion of Setdb1 exon 3 was verified by PCR from FACS-sorted GFP^{+} cells of Tamoxifen-treated Lgr5-GFP-Cre^{ERT2} and Lgr5-GFP-Cre^{ERT2}/ Setdb1$^{lox/lox}$ (E) or Lgr5-GFP-Cre^{ERT2}/nTnG and Lgr5-GFP-Cre^{ERT2}/ Setdb1$^{lox/lox}$/nTnG (F) mice, using a primer pair hybridizing to sequences inside Exon 3 (F) and outside the LoxP site (R). The data from 4 cell preparations and mean their values are presented as relative to the values obtained with cells from Lgr5-GFP-Cre^{ERT2} and Lgr5-GFP-Cre^{ERT2}/nTnG mice.

(G) Representative images of intestinal organoids from FACS-sorted GFP^{+} cells, isolated from two Lgr5-GFP-Cre^{ERT2}/nTnG mice (upper panel) and two Lgr5-GFP-Cre^{ERT2}/Setdb1$^{lox/lox}$/nTnG mice (bottom panel). After seeding, the cells were treated with 100nM tamoxifen (TAM) for 10 days, before image acquisition. Images from organoids developed from GFP^{+} cells isolated from two different mice (biological replicates) are shown. OF (Organoid Formation) values correspond to the number of fully developed, GFP-labeled, complex organoids with distinguishable crypt-like domains from 1000 seeded GFP^{+} cells at day10. Numbers from 3 parallel seedings (technical replicates) are shown.

(H) Representative images of 2nd passage organoids originating from tamoxifen-treated Lgr5-GFP-Cre^{ERT2}/nTnG organoids (upper panel) and from Lgr5-GFP-Cre^{ERT2}/ Setdb1$^{lox/lox}$/nTnG organoids (bottom panel). Images were taken 10 days after reseeding the cells.

Figure S2

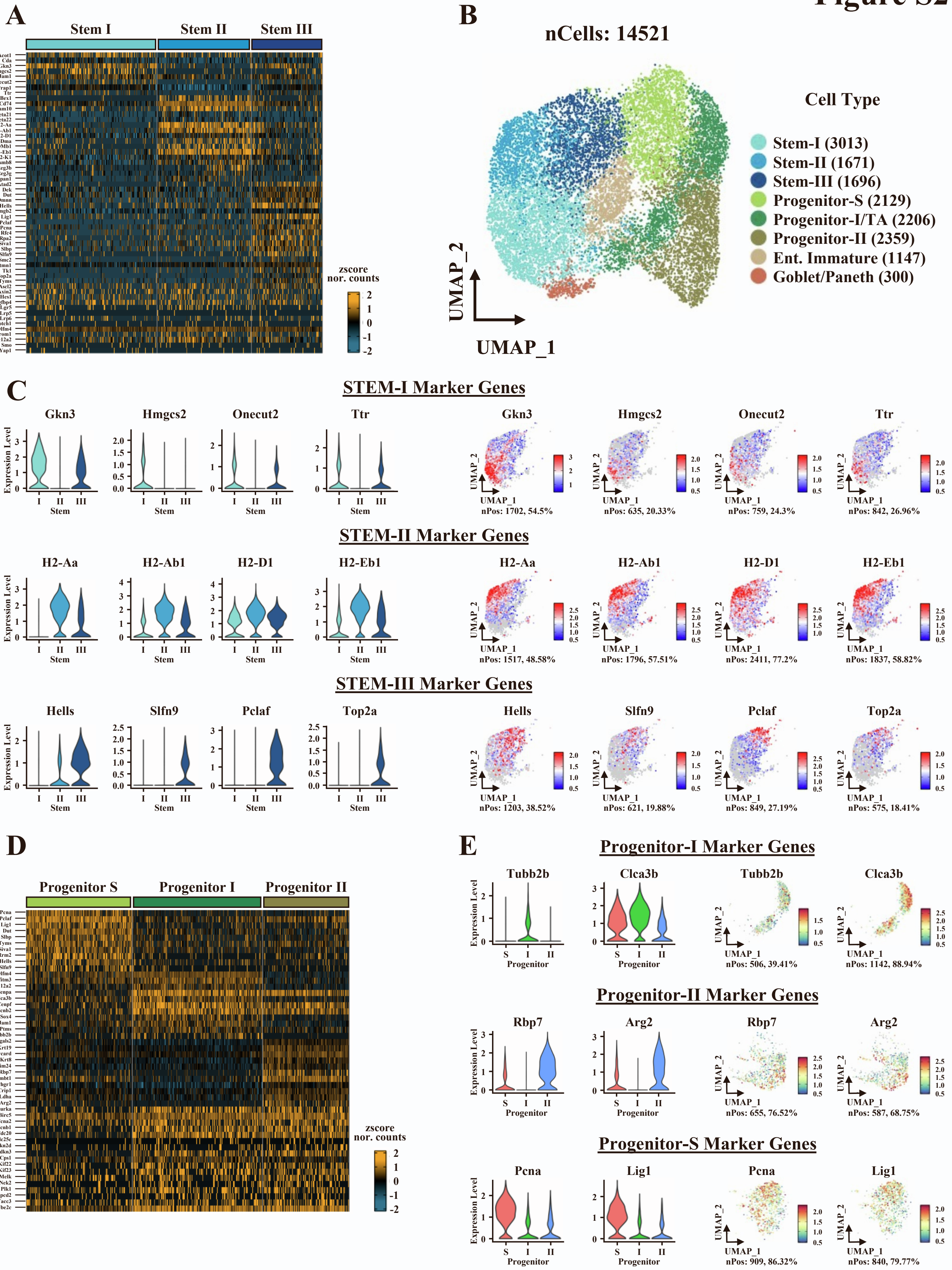
A
Stem I
Stem II
Stem III
Acot1
Cda
Gkn3
Hmgcs2
Jam1
Onecut2
Prap1
Ttr
Bex1
Cd74
Ceacam10
Defa21
Defa22
H2-Aa
H2-Ab1
H2-D1
H2-Dma
H2-DMb1
H2-Eb1
H2-K1
Psmb8
Reg3b
Reg3g
Tspan1
Atad2
Dek
Dut
Dnmt1
Hells
Hmgb2
Lig1
Pclaf
Pcna
Rfc4
Rpa2
Siva1
Slbp
Slfn9
Smc2
Stmn1
Tk1
Top2a
Tyms
Ascl2
Axin2
Hes1
Igfbp4
Lgr5
Lrp5
Lrp6
Notch1
Olfm4
Prom1
Slc12a2
Smo
Yap1
zscore
nor. counts
B
nCells: 14521
UMAP_2
UMAP_1
Cell Type
Stem-I (3013)
Stem-II (1671)
Stem-III (1696)
Progenitor-S (2129)
Progenitor-I/TA (2206)
Progenitor-II (2359)
Ent. Immature (1147)
Goblet/Paneth (300)
C
STEM-I Marker Genes
Gkn3
Hmgcs2
Onecut2
Ttr
Expression Level
Stem
nPos: 1702, 54.5%
nPos: 635, 20.33%
nPos: 759, 24.3%
nPos: 842, 26.96%
STEM-II Marker Genes
H2-Aa
H2-Ab1
H2-D1
H2-Eb1
nPos: 1517, 48.58%
nPos: 1796, 57.51%
nPos: 2411, 77.2%
nPos: 1837, 58.82%
STEM-III Marker Genes
Hells
Slfn9
Pclaf
Top2a
nPos: 1203, 38.52%
nPos: 621, 19.88%
nPos: 849, 27.19%
nPos: 575, 18.41%
D
Progenitor S
Progenitor I
Progenitor II
Pcna
Pclaf
Lig1
Dut
Slbp
Tyms
Siva1
Rrm2
Hells
Slfn9
Olfm4
Ifitm3
Slc12a2
Cenpa
Clca3b
Cenpf
Ccnb2
Sox4
Jam1
Ptms
Tubb2b
Lgals2
Krt19
Pycard
Krt8
Smim24
Rbp7
Dmbt1
Phgr1
Crip1
Ldha
Arg2
Aurka
Birc5
Ccna2
Ccnb1
Cdc20
Cdc25c
Cdkn2d
Cdkn3
Cps1
Kif22
Kif23
Melk
Nek2
Plk1
Sapcd2
Tacc3
Ube2c
zscore
nor. counts
E
Progenitor-I Marker Genes
Tubb2b
Clca3b
Progenitor
nPos: 506, 39.41%
nPos: 1142, 88.94%
Progenitor-II Marker Genes
Rbp7
Arg2
nPos: 655, 76.52%
nPos: 587, 68.75%
Progenitor-S Marker Genes
Pcna
Lig1
nPos: 909, 86.32%
nPos: 840, 79.77%

Figure S2
Gene signatures characterizing Stem cell and Progenitor cell subsets. Related to Figure 2.

(A) Heatmap of relative mRNA levels (zscore normalized counts) of genes in Stem-I, Stem-II and Stem-III cell clusters.

(B) UMAP projection of Stem cell and Progenitor cell types after sub-clustering of the indicated cell types. The color codes of the resulting re-clustered cell types are shown at the right.

(C) Violin plots and Feature plots of selected marker genes characterizing Stem cell subpopulations.

(D) Heatmap of relative mRNA levels (zscore normalized counts) of genes in Progenitor-I and Progenitor-II cell clusters. The gene expression profile of Progenitor S cells indicate that this cluster represents Progenitor cells in the S phase of the cell cycle.

(E) Violin plots and Feature plots of selected marker genes characterizing Progenitor cell subpopulations.

Figure S3

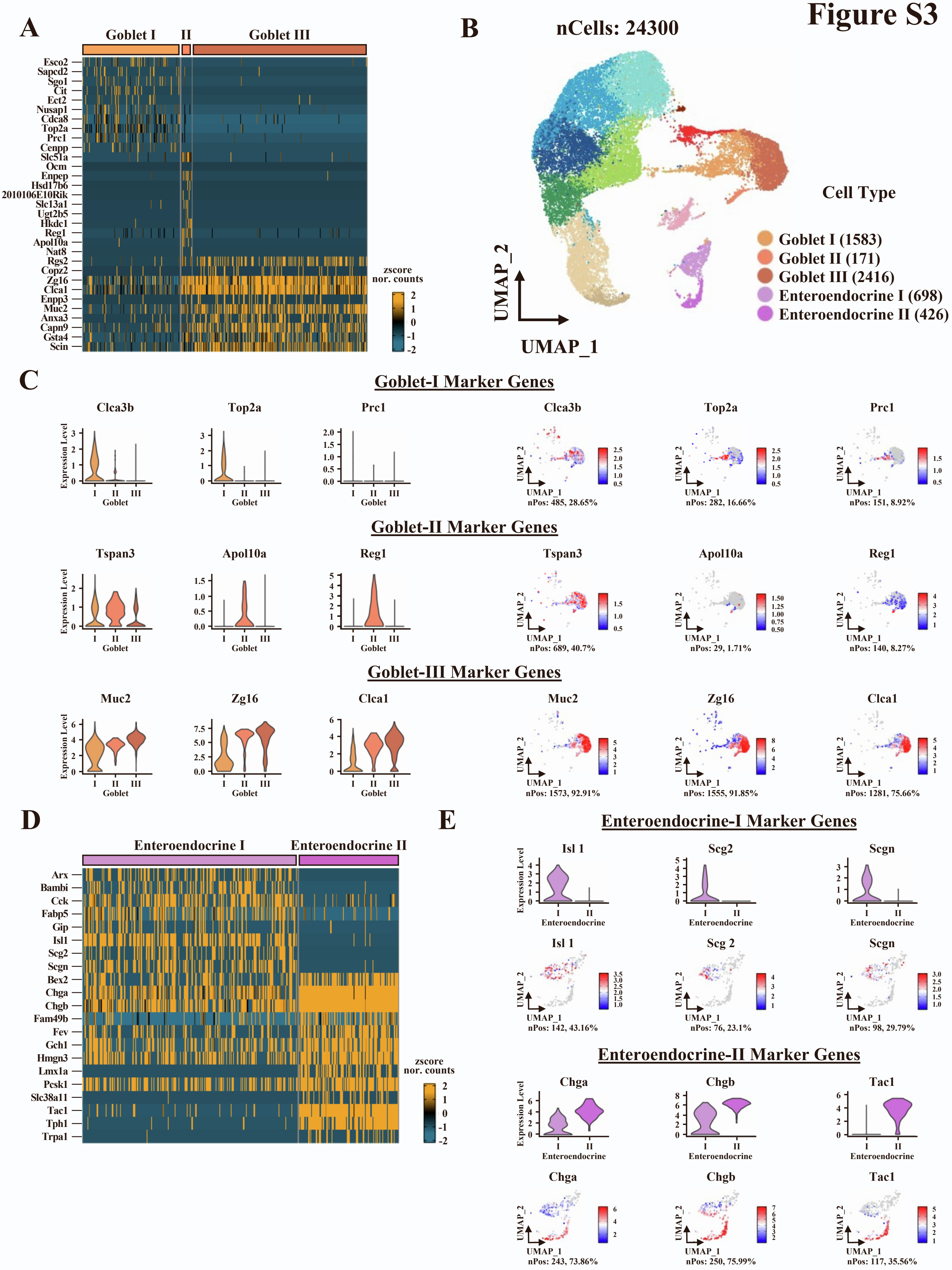
A
Goblet I
II
Goblet III
Esco2
Sapcd2
Sgo1
Cit
Ect2
Nusap1
Cdca8
Top2a
Prc1
Cenpp
Slc51a
Ocm
Enpep
Hsd17b6
2010106E10Rik
Slc13a1
Ugt2b5
Hkdc1
Reg1
Apol10a
Nat8
Rgs2
Copz2
Zg16
Clca1
Enpp3
Muc2
Anxa3
Capn9
Gsta4
Scin
zscore
nor. counts
B
nCells: 24300
Cell Type
Goblet I (1583)
Goblet II (171)
Goblet III (2416)
Enteroendocrine I (698)
Enteroendocrine II (426)
UMAP_2
UMAP_1
C
Goblet-I Marker Genes
Clca3b
Top2a
Prc1
Expression Level
Goblet
nPos: 485, 28.65%
nPos: 282, 16.66%
nPos: 151, 8.92%
Goblet-II Marker Genes
Tspan3
Apol10a
Reg1
nPos: 689, 40.7%
nPos: 29, 1.71%
nPos: 140, 8.27%
Goblet-III Marker Genes
Muc2
Zg16
Clca1
nPos: 1573, 92.91%
nPos: 1555, 91.85%
nPos: 1281, 75.66%
D
Enteroendocrine I
Enteroendocrine II
Arx
Bambi
Cck
Fabp5
Gip
Isl1
Scg2
Scgn
Bex2
Chga
Chgb
Fam49b
Fev
Gch1
Hmgn3
Lmx1a
Pcsk1
Slc38a11
Tac1
Tph1
Trpa1
zscore
nor. counts
E
Enteroendocrine-I Marker Genes
Isl 1
Scg2
Scgn
Enteroendocrine
Isl 1
Scg 2
Scgn
nPos: 142, 43.16%
nPos: 76, 23.1%
nPos: 98, 29.79%
Enteroendocrine-II Marker Genes
Chga
Chgb
Tac1
Chga
Chgb
Tac1
nPos: 243, 73.86%
nPos: 250, 75.99%
nPos: 117, 35.56%

Figure S3
Gene signatures characterizing Goblet cell and Enteroendocrine cell subsets. Related to Figure 2.

(A) Heatmap of relative mRNA levels (z-score normalized counts) of genes in Goblet-I, Goblet-II and Goblet-III cell clusters.

(B) UMAP projection of cell types from Figure 2A, used to characterize Goblet and Enteroendocrine cell subtypes.

(C) Violin plots and Feature plots of selected marker genes characterizing Goblet cell subpopulations.

(D) Heatmap of relative mRNA levels (z-score normalized counts) of genes in Enteroendocrine-I (EEC-I) and Enteroendocrine-II (EEC-II) cell clusters.

(E) Violin plots and Feature plots of selected marker genes characterizing Enteroendocrine cell subpopulations.

Figure S4

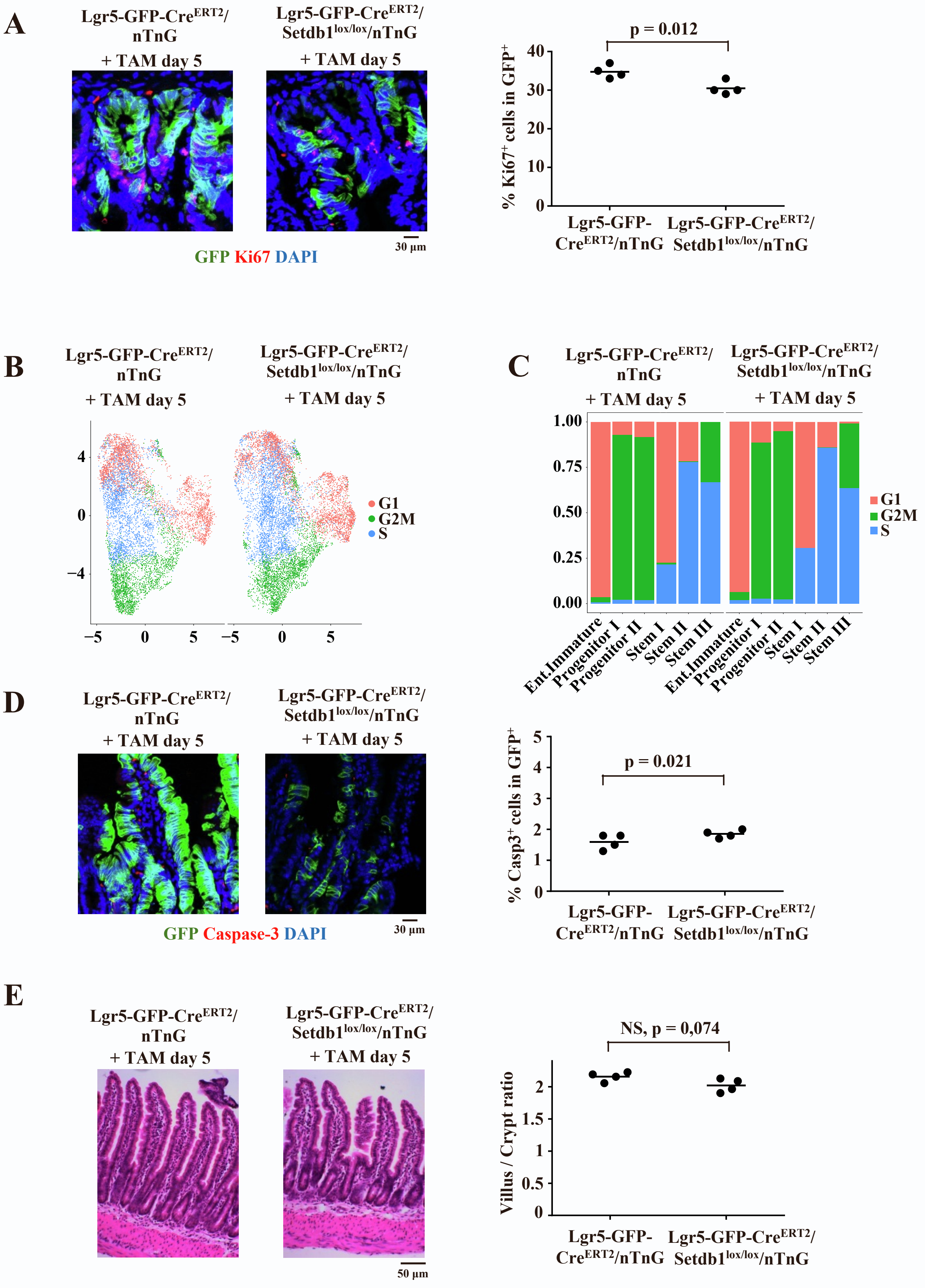
A
Lgr5-GFP-CreERT2/ nTnG + TAM day 5
Lgr5-GFP-CreERT2/ Setdb1lox/lox/nTnG + TAM day 5
30 µm
GFP Ki67 DAPI
p = 0.012
% Ki67+ cells in GFP+
Lgr5-GFP-CreERT2/nTnG
Lgr5-GFP-CreERT2/ Setdb1lox/lox/nTnG
B
Lgr5-GFP-CreERT2/ nTnG + TAM day 5
Lgr5-GFP-CreERT2/ Setdb1lox/lox/nTnG + TAM day 5
G1
G2M
S
C
Lgr5-GFP-CreERT2/ nTnG + TAM day 5
Lgr5-GFP-CreERT2/ Setdb1lox/lox/nTnG + TAM day 5
Ent.Immature
Progenitor I
Progenitor II
Stem I
Stem II
Stem III
G1
G2M
S
D
Lgr5-GFP-CreERT2/ nTnG + TAM day 5
Lgr5-GFP-CreERT2/ Setdb1lox/lox/nTnG + TAM day 5
30 µm
GFP Caspase-3 DAPI
p = 0.021
% Casp3+ cells in GFP+
Lgr5-GFP-CreERT2/nTnG
Lgr5-GFP-CreERT2/ Setdb1lox/lox/nTnG
E
Lgr5-GFP-CreERT2/ nTnG + TAM day 5
Lgr5-GFP-CreERT2/ Setdb1lox/lox/nTnG + TAM day 5
50 µm
NS, p = 0,074
Villus / Crypt ratio
Lgr5-GFP-CreERT2/nTnG
Lgr5-GFP-CreERT2/ Setdb1lox/lox/nTnG

Figure S4.

Analysis of cell proliferation and apoptosis in Lgr5-GFP-Cre^{ERT2}/ Setdb1$^{lox/lox}$/nTnG cells 5 days after tamoxifen treatment. Related to Figure 4.

(A) Immunostaining with Ki67 antibody in cryosections from ileal tissue of Lgr5-GFP-Cre^{ERT2}/nTnG and Lgr5-GFP-Cre^{ERT2} /Setdb1$^{lox/lox}$/nTnG mice 5 days after tamoxifen treatment. The graph at right shows percentages of Ki67/GFP double positive cells identified in GFP$^+$ cells counted in 30 villus-crypt areas from individual mice (n=4). p-values were determined by Student's t-test. Note: Counting only GFP$^+$ cells warrant the evaluation of cells originating from Lgr5+ cells where Setdb1 is inactivated.

(B) UMAP projection of single cells from Tamoxifen-treated Lgr5-GFP-Cre^{ERT2}/nTnG and Lgr5-GFP-Cre^{ERT2} /Setdb1$^{lox/lox}$/nTnG mice based on the expression of cell cycle marker genes.

(C) Relative distribution of G1, S and G2/M-phase cells in the individual cell clusters in Tamoxifen-treated Lgr5-GFP-Cre^{ERT2}/nTnG and Lgr5-GFP-Cre^{ERT2} /Setdb1$^{lox/lox}$/nTnG mice.

(D) Immunostaining with Caspase-3 antibody in cryosections from ileal tissue of Lgr5-GFP-Cre^{ERT2}/nTnG and Lgr5-GFP-Cre^{ERT2} /Setdb1$^{lox/lox}$/nTnG mice 5 days after tamoxifen treatment. The graph at right shows percentages of Caspase-3/GFP double positive cells identified in GFP+ cells counted in 30 villus-crypt areas from individual mice (n=4). p-values were determined by Student's t-test.

(E) Representative pictures of Hematoxylin and Eosin (H&E)-stained paraffin-embedded ileal tissues from Lgr5-GFP-Cre^{ERT2}/nTnG and Lgr5-GFP-Cre^{ERT2} /Setdb1$^{lox/lox}$/nTnG mice 5 days after tamoxifen treatment. Villus-crypt ratios were calculated from 50 ileal areas in individual mice (n=4). p-values were determined by Student's t-test.

Figure S5

A

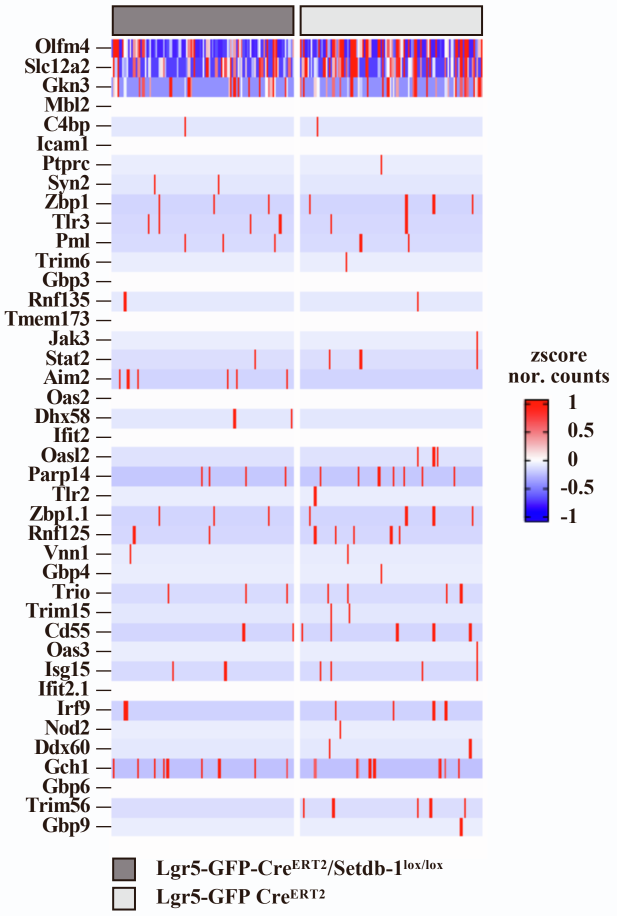

Olfm4
Slc12a2
Gkn3
Mbl2
C4bp
Icam1
Ptprc
Syn2
Zbp1
Tlr3
Pml
Trim6
Gbp3
Rnf135
Tmem173
Jak3
Stat2
Aim2
Oas2
Dhx58
Ifit2
Oasl2
Parp14
Tlr2
Zbp1.1
Rnf125
Vnn1
Gbp4
Trio
Trim15
Cd55
Oas3
Isg15
Ifit2.1
Irf9
Nod2
Ddx60
Gch1
Gbp6
Trim56
Gbp9
zscore
nor. counts
1
0.5
0
-0.5
-1
Lgr5-GFP-CreERT2/Setdb-1lox/lox
Lgr5-GFP CreERT2

B

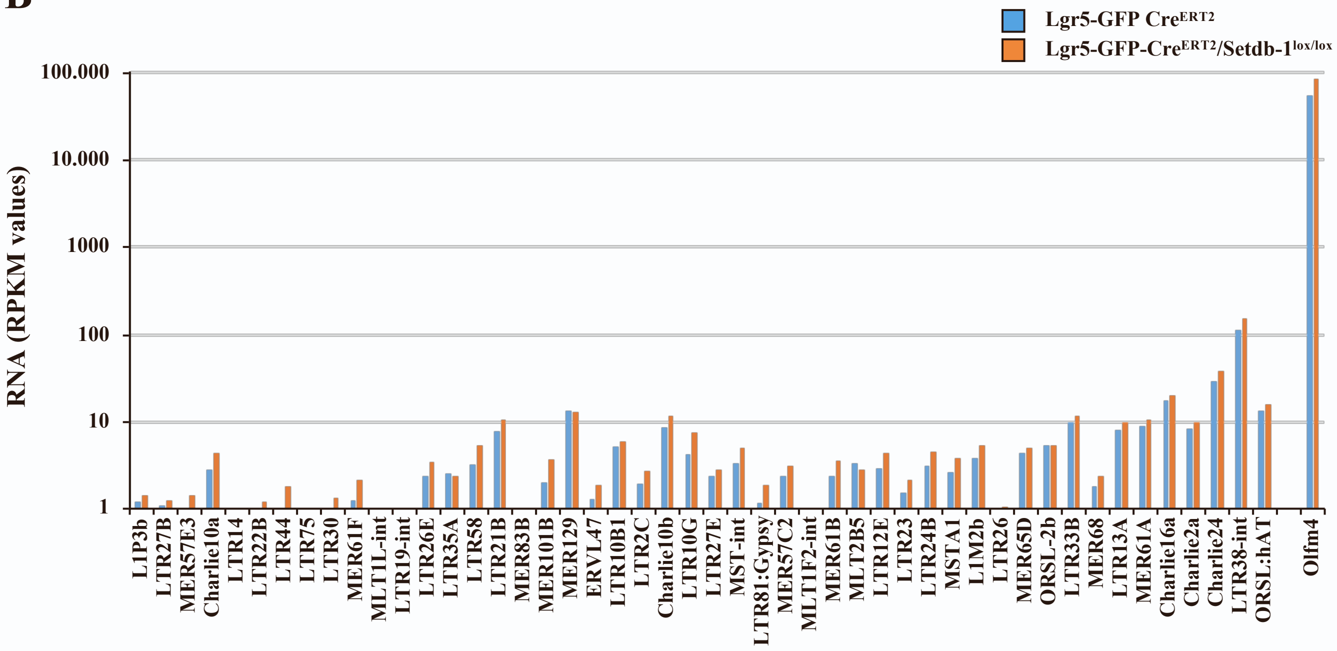

Lgr5-GFP CreERT2
Lgr5-GFP-CreERT2/Setdb-1lox/lox
RNA (RPKM values)
100.000
10.000
1000
100
10
1
L1P3b
LTR27B
MER57E3
Charlie10a
LTR14
LTR22B
LTR44
LTR75
LTR30
MER61F
MLT1L-int
LTR19-int
LTR26E
LTR35A
LTR58
LTR21B
MER83B
MER101B
MER129
ERVL47
LTR10B1
LTR2C
Charlie10b
LTR10G
LTR27E
MST-int
LTR81:Gypsy
MER57C2
MLT1F2-int
MER61B
MLT2B5
LTR12E
LTR23
LTR24B
MSTA1
L1M2b
LTR26
MER65D
ORSL-2b
LTR33B
MER68
LTR13A
MER61A
Charlie16a
Charlie2a
Charlie24
LTR38-int
ORSL:hAT
Olfm4

Figure S5

Lack of activation of innate immunity genes and endogenous retrovirus activation in Lgr5-GFP-Cre^{ERT2}/ Setdb1$^{lox/lox}$ cells 5 days after tamoxifen induction. Related to Figure 2.

(A) Heatmap showing RNA levels of innate immunity genes in individual cells from tamoxifen-treated Lgr5-GFP-Cre^{ERT2} (WT, light gray) and Lgr5-GFP-Cre^{ERT2}/ Setdb1$^{lox/lox}$ (Setdb1-KO, dark gray) mice. Heatmap of the highly expressed Olfm4 is included in the top panel.

(B) Bar-graph showing relative mRNA levels of the indicated endogenous retroviral transcripts in FACS-sorted GFP^{+} cells from tamoxifen-treated Lgr5-GFP-Cre^{ERT2} mice and Lgr5-GFP-Cre^{ERT2}/ Setdb1$^{lox/lox}$ mice, as determined Tettranscript analysis of total RNA-seq data. Bars represent average RPKM values from two biological replicates. For comparison Olfm4 mRNA levels are included in the last bars. Note the 3 to 4 orders of magnitude difference between the retroviral RNAs and Olfm4 RNA levels and the small or no difference between the values obtained with wild type and Setdb1-deficient cells.

Figure S6

A

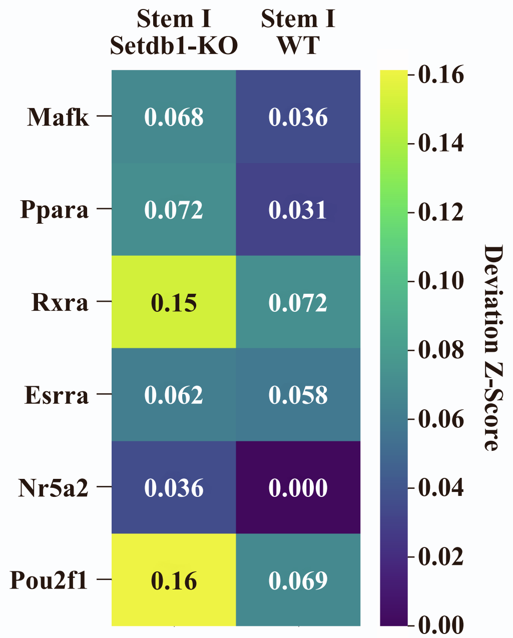

B

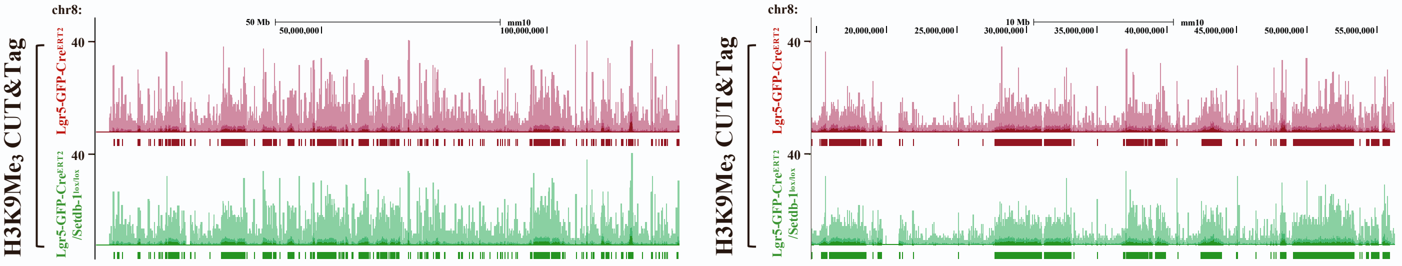

C

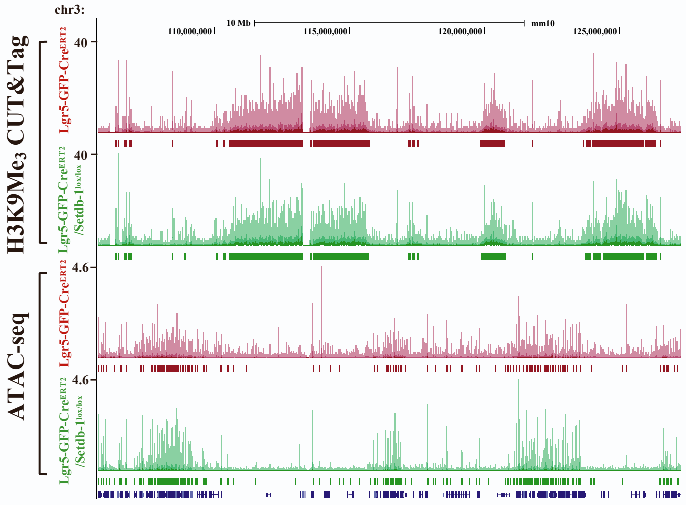

Figure S6

Comparisons of specific Transcription Factor Regulons and large H3K9Me$_3$-modified heterochromatin domains in wild-type and Setdb1-deficient cells. Related to Figure 5.

(A) Transcription Factor Regulons for Mafk, Ppara, Rxra, Esrra and Pouf1were compared using the PyScenic computational pipeline (Ref). Numbers inside the heatmaps correspond to mean AUC (Area Under Curve) scores of the indicated regulons, after ten runs of the Pyscenic algorithm on the scRNA-seq dataset of Stem-I cluster for either wild type (TAM-treated Lgr5-GFP-Cre^{ERT2}) or Setdb1-KO (TAM-treated Lgr5-GFP-Cre^{ERT2}/ Setdb1$^{lox/lox}$) conditions separately.
p-adjusted values for the different factors were as follows:
Pou2f1(+) 0.0; Rxra(+) 3.291e^{-179}; Ppara(+) 2.255e^{-119}; Mafk(+) 4.802e^{-107}; Esrra(+) 0.999. Note that with the exception of Esrra, Setdb1 inactivation, significantly affected the downstream regulons of the interrogated Transcription Factors. For the Nr5a2 (+) we couldn't provide a statistical test, since it never appeared as an active regulon on the wildtype Stem-I cluster.

(B) Limited changes in large H3K9Me$_3$-modified heterochromatin domains in Setdb1-deficient cells. Genome Browser tracks showing normalized H3K9Me$_3$ reads and SICER-called peaks (bars shown below the tracks) along the entire chromosome 8 (left panel) and a 40 Mb region of chromosome 8, from tamoxifen-treated Lgr5-GFP-Cre^{ERT2} mice (WT) and Lgr5-GFP-Cre^{ERT2}/ Setdb1$^{lox/lox}$ mice (Setdb1-KO). Note the highly similar overall distribution of the peak locations especially the broad peaks.

(C) Combined Genome Browser tracks of H3K9Me$_3$ CUT&Tag and ATAC-seq reads spanning a 20Mb region of chromosome 3. Note the tandemly organized H3K9Me$_3$-containing heterochromatin domains and the ATAC-seq signal containing euchromatin domains. Note the rare co-occurrence of ATAC-seq peaks and broad H3K9Me$_3$ CUT&Tag peaks in cells from Lgr5-GFP-Cre^{ERT2} mice, and their more frequent co-occurrence in Setdb1-KO cells.

Figure S7

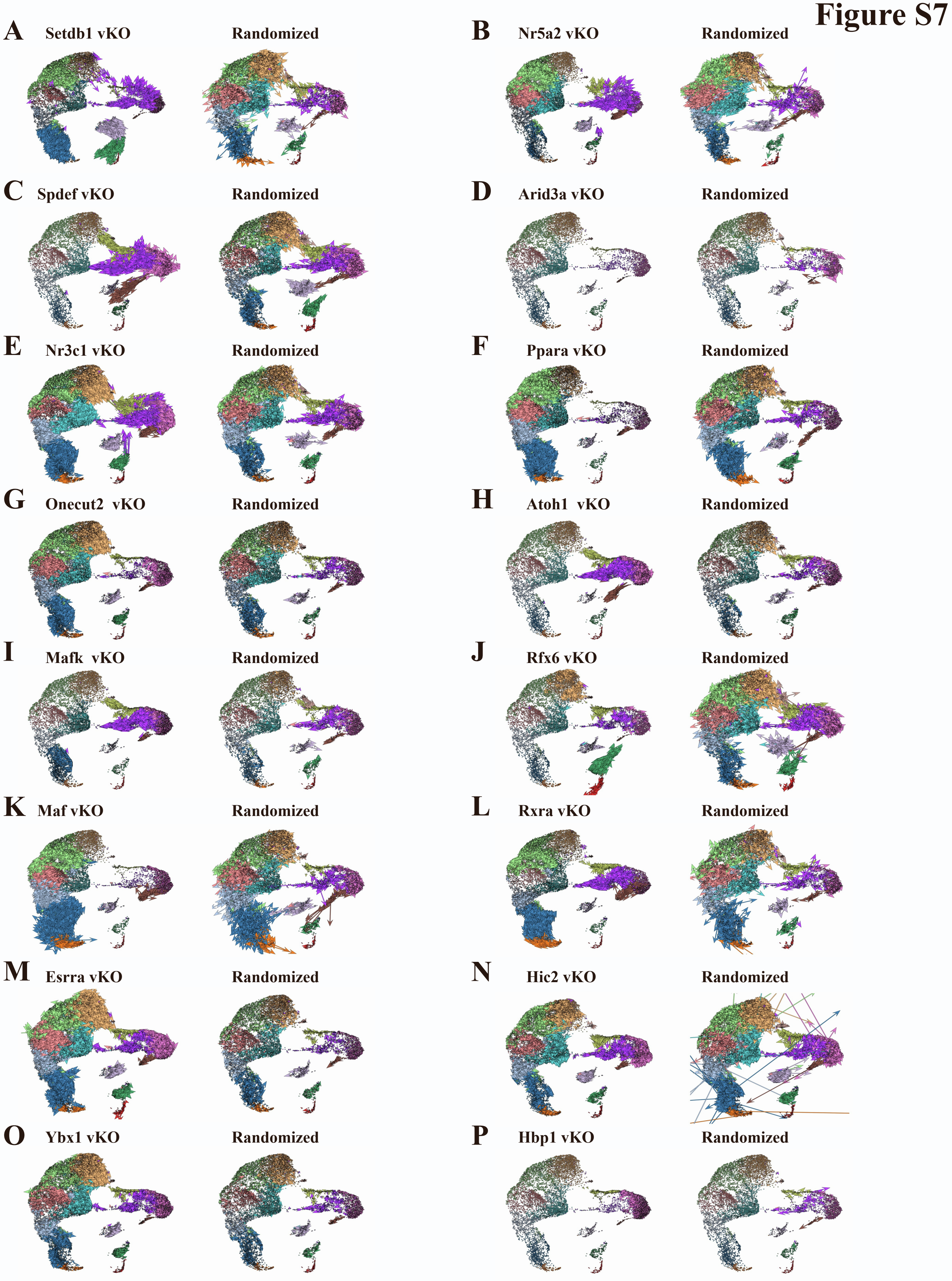
A Setdb1 vKO
Randomized
B Nr5a2 vKO
Randomized
C Spdef vKO
Randomized
D Arid3a vKO
Randomized
E Nr3c1 vKO
Randomized
F Ppara vKO
Randomized
G Onecut2 vKO
Randomized
H Atoh1 vKO
Randomized
I Mafk vKO
Randomized
J Rfx6 vKO
Randomized
K Maf vKO
Randomized
L Rxra vKO
Randomized
M Esrra vKO
Randomized
N Hic2 vKO
Randomized
O Ybx1 vKO
Randomized
P Hbp1 vKO
Randomized

Figure S7

Quality control of in-silico perturbation experiments virtual Setdb1 and fifteen transcription factors. Related to Figure 7.

(A-P) CellOracle machine-learning algorithm[40] was employed to simulate the virtual knockout (vKO) of the transcription factors (TFs) across the single-cell manifold. Each transcription factor has two panels. Left panel shows UMAP plot displaying the predicted vector field of cell state shifts following specific TF deletion. The direction and length of the arrows represent the predicted trajectory and magnitude of transcriptomic change, respectively, for individual cells. Right panels show randomized control simulations where the regulatory network edges were shuffled, demonstrating that the directional flow observed in the specific vKO is specific to the inferred gene regulatory network. Colors represent distinct cell type clusters.

Figure S8

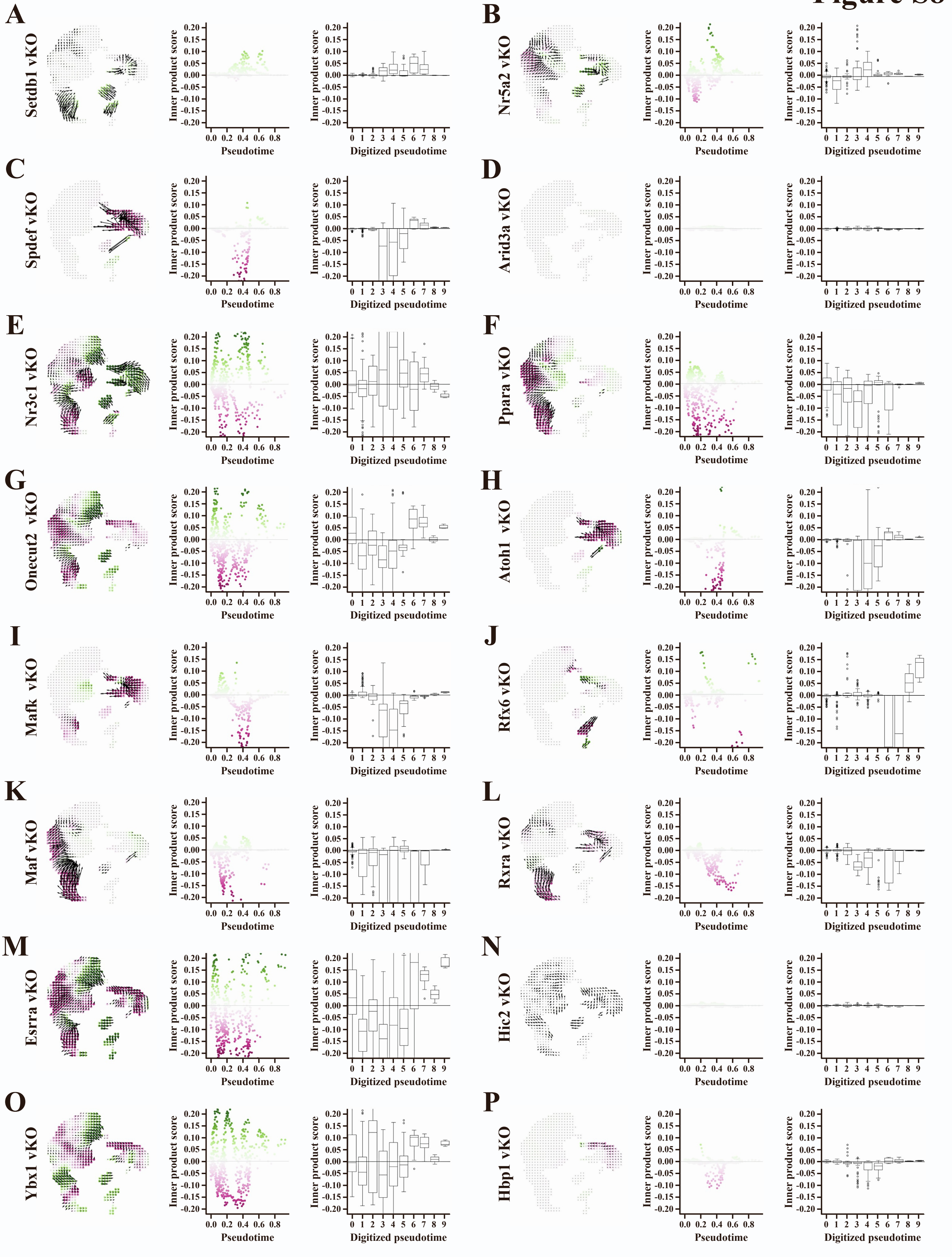

A
Setdb1 vKO
B
Nr5a2 vKO
C
Spdef vKO
D
Arid3a vKO
E
Nr3c1 vKO
F
Ppara vKO
G
Onecut2 vKO
H
Atoh1 vKO
I
Mafk vKO
J
Rfx6 vKO
K
Maf vKO
L
Rxra vKO
M
Esrra vKO
N
Hic2 vKO
O
Ybx1 vKO
P
Hbp1 vKO
Inner product score
0.20
0.15
0.10
0.05
0.00
-0.05
-0.10
-0.15
-0.20
0.0 0.2 0.4 0.6 0.8
Pseudotime
0 1 2 3 4 5 6 7 8 9
Digitized pseudotime

Figure S8. Quantitative comparison of CellOracle perturbation flows with the endogenous developmental program. Related to Figure 7.

Inner products between CellOracle-predicted perturbation vectors and the baseline developmental flow were calculated across the UMAP manifold for the indicated virtual knockouts (vKOs). Left panels, perturbation vector field overlaid with inner-product values (green, positive; magenta, negative). Middle panels, inner-product scores plotted against continuous pseudotime. Panels at right, boxplots of inner-product scores across digitized pseudotime bins.

Figure S9

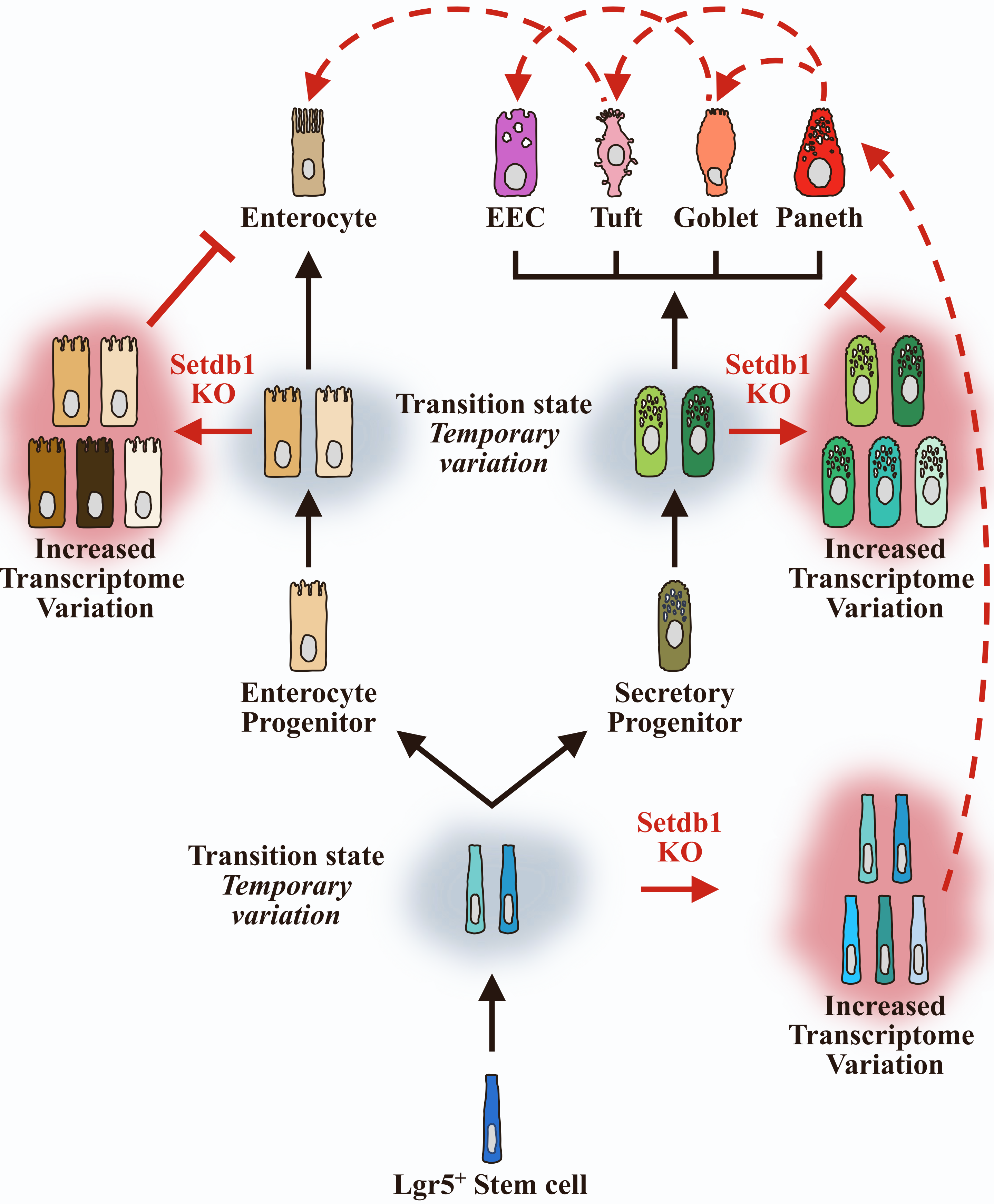

Enterocyte
EEC
Tuft
Goblet
Paneth
Setdb1 KO
Transition state
Temporary variation
Setdb1 KO
Increased Transcriptome Variation
Increased Transcriptome Variation
Enterocyte Progenitor
Secretory Progenitor
Setdb1 KO
Transition state
Temporary variation
Increased Transcriptome Variation
Lgr5+ Stem cell

Figure S9

Model of chromatin defect-mediated increased cell-to-cell transcriptome variation during intestinal stem cell differentiation.

The model proposes a multilevel control mechanism for the temporally distinct transcriptomes, at each consecutive differentiation state. Tight regulation promotes relatively uniform cellular transcriptomes, which is characteristic to Stem cells, Enterocyte Progenitor and Secretory Progenitors, as well as the differentiated epithelial cell types (Enterocytes, Paneth, Goblet, Tuft and Enteroendocrine cells). During the transition between the consecutive cellular differentiation states, partial transcriptome diversification occurs, leads to transition state-specific variable transcriptomes (cells in gray clouds). In Setdb1-KO mice, defects in chromatin structure-dependent regulation block cells in the transition states further increasing cell-to-cell transcriptional variations (cells in pink clouds), which cannot progress to fully differentiated cell types (flat-ended bar). Alternative pathways for the generation of secretory cell types directly from Stem cell population are triggered (dashed red arrow).
